# London Trauma Conference 2015: London, UK, 8-11 December 2015

**DOI:** 10.1186/s13049-016-0248-x

**Published:** 2016-06-17

**Authors:** Pascale Avery, Leopold Salm, Flora Bird, Anja Hutchinson, Ashley Matthies, Anthony Hudson, Heather Jarman, Maria Bergman Nilsson, Tom Konig, Nigel Tai, Espen Fevang, Børge Hognestad, Håkon B. Abrahamsen, Olivia V. Cheetham, Matthew J. C. Thomas, Kieron D. Rooney, Josephine Murray, Malcolm Tunnicliff, Joseph W. Collinson, Thomas Brown, Christopher Pritchett, Christopher S. A. Pritchett, Mark Jadav, Gareth Meredith, Jamie Plumb, Steve Harris, Roger Langford, J. G. Hunter, A. Sage, R. Madden, O. Flamank, B. Broadbent, S. Marsh, H. Lewis, E. Daniels, N. Roberts, J. G. Hunter, A. Sage, R. Madden, O. Flamank, B. Broadbent, S. Marsh, H. Lewis, E. Daniels, N. Lin, N. Roberts, Samuel Bulford, Silas Houghton-Budd, Sam Pearson, Megan Clear-Hill, David J. Menzies, James P. Leonard, Conor Keogh, Ray Quinn, John D. Hinds, N. Roberts, D. Ashton-Cleary, M. Jadav, Ismail Mahmood, Ayman El-Menyar, Basil Younis, Ahmed Khalid, Syed Nabir, Mohamed Nadeem Ahmed, Omer Al-Yahri, Hassan Al-Thani, Katie Young, Susan A. Hendrickson, Georgina Phillips, Matthew D. Gardiner, Shehan Hettiaratchy, Alexandra Alice Crossland, Anthony Hudson, Nicholas C. Brassington, Anthony Hudson, Emily McWhirter, Bjørn O. Reid, Marius Rehn, Oddvar Uleberg, Andreas J. Krüger, Cara Jennings, Yasmin Kapadia, Duncan Bew, Jenny Townsend, Tom P. Hurst, Elizabeth A. Foster, Thomas B. Brown, Joseph Collinson, Christopher Pritchett, Toby Slade, Kristin Tønsager, Marius Rehn, Kjetil G.Ringdal, Andreas J.Krüger, Rasmus Hesselfeldt, Sandra Wulffeld, Asger Sonne, Lars S. Rasmussen, Jacob Steinmetz, Thomas J. Renninson, Nadine Thomson, Harvey Pynn, Timothy J. Hooper, Anthony Hudson, Jacinta Dawson, Ashley Matthies, Morten Langfeldt Friberg, Leif Rognås, Jessica F. G. Wills, Anthony Hudson, Conor D. A. Turner, Marius Rehn, James Nunn, Mete Erdogan, Robert S. Green, Samuel Minor, Mete Erdogan, Kathy Hartlen, Robert S. Green, Ruth Bird, Rachael L. Grupping, Amelia M. Stacey, Marius Rehn, David J. Lockey, S. Abiks, L. Cutler, K. Monaghan, A. Al-Rais, C. Hymers, R. Bloomer, Y. Kapadia, Sophie-Charlott Seidenfaden, Ingunn S. Riddervold, Hans Kirkegaard, Niels Juul, Morten T. Bøtker, Alice Gao, Zane Perkins, Gareth Grier, Alex Tzannes, Nathan J. Hudson-Peacock, Quentin Otto, Laurie Phillipson, Rik Thomas, Ainsley Heyworth, Quentin Otto, Nathan J. Hudson-Peacock, Laurie Phillipson, Ainsley Heyworth, Erica Ley, Daniel Banner, Ainsley Heyworth, Erica Ley, Madeleine Benson, Nathan Hudson-Peacock, Tony Stone, Erica Ley, Louise Rousson, Ainsley Heyworth, Beth A. Lineham, Matthew J. Lee, Martin Gough, William H. Seligman, Hannah E. Thould, Andrew Dinsmore, Charlotte Tan, Julian Thompson, C. Andy Eynon, David J. Lockey, Rebecka M. Rubenson Wahlin, Veronica Lindström, Sari Ponzer, Veronica Vicente, Pamela Eligio, Anthony Hudson, Robert Young, Dimitri Amiras, Ian Sinha

**Affiliations:** The Royal Marsden Hospital, Chelsea, London, UK; The Royal London Hospital, London, UK; The Royal Free Hospital, London, UK; Emergency Department, St. Georges University Hospital, London, UK; Queen Mary University of London, Barts and The London School of Medicine and Dentistry, Whitechapel, London, UK; Dept of Trauma Surgery, Barts Health NHS Trust, Whitechapel High St, London, E1 1BB UK; Department of Research and Development, Norwegian Air Ambulance Foundation, Drøbak, Norway; Department of Anaesthesiology and Intensive Care, Stavanger University Hospital, Stavanger, Norway; University Hospitals Bristol, Bristol, UK; University Hospitals Bristol, Bristol, UK; Great Western Air Ambulance, South West Ambulance Service Trust, Exeter, UK; Emergency Department, King’s College Hospital, London, UK; Emergency Department, Royal Cornwall Hospital, Truro, UK; Simulation Department, Royal Cornwall Hospital, Truro, UK; Emergency Department, Royal Cornwall Hospital (RCH), Truro, UK; Peninsula School of Medical and Dentistry, Royal Cornwall Hospital (RCH), Truro, UK; Emergency Department, Royal Cornwall Hospital (RCH), Truro, UK; Peninsula School of Medical and Dentistry, Royal Cornwall Hospital (RCH), Truro, UK; Department of Mathematics and Information Sciences, Northumbria University, Newcastle upon Tyne, UK; Medical School, University College London, London, UK; Ashford make-ready station, South East Coast Ambulance Service, Ashford, UK; Motorcycling Ireland Medical Team, Dublin, Ireland; Emergency Department St Vincent’s University Hospital, Dublin, Ireland; Royal Cornwall Hospital, Truro, UK; Department of Surgery, Trauma Surgery, Hamad General Hospital, Doha, Qatar; Major Trauma Centre, St Marys Hospital, Imperial College NHS Healthcare Trust, London, UK; Imperial College, London, UK; St George’s Accident and Emergency Department, St George’s Hospital Medical School (University of London), Tooting, London, UK; St George’s University of London, London, UK; Emergency Department, St George’s Hospital, London, UK; Kent Surrey and Sussex Air Ambulance Trust Helicopter Emergency Medical Service (KSSAAT HEMS), Redhill, UK; Department of Emergency Medicine and Prehospital Services, St. Olavs Hospital, Trondheim, Norway; Department of Research and Development, Norwegian Air Ambulance Foundation, Drøbak, Norway; London’s Air Ambulance, Barts Health Trust, Whitechapel, London, UK; Field of Pre-hospital Critical Care, Network of Medical Sciences, University of Stavanger, Stavanger, Norway; Emergency Department, St. Thomas’ Hospital, London, UK; Emergency Department, King’s College Hospital, London, UK; Trauma and Emergency Surgery, King’s College Hospital, London, UK; Helicopter Emergency Medical Service, Barts Health NHS Trust, London, UK; Emergency Department, Royal Cornwall Hospital, Truro, UK; The Norwegian Air Ambulance Foundation, Drøbak, Norway; Department of Anaesthesiology and Intensive Care, Stavanger University Hospital, Stavanger, Norway; London’s Air Ambulance, Barts Health, London, UK; Department of Anaesthesiology, Vestfold Hospital Trust, Tønsberg, Norway; Department of Anaesthesia and Acute Care, St. Olavs Hospital, Trondheim, Norway; Department of Anaesthesia, Centre of Head and Orthopaedics, Rigshospitalet, University of Copenhagen, Copenhagen, Denmark; Department of Anaesthetics, Gloucester Royal Hospital, Gloucester, UK; Great Western Air Ambulance, Filton Air Support Unit, Bristol, UK; Intensive Care Department, Southmead Hospital, Bristol, UK; Emergency Department, St George’s Hospital, London, London, UK; Kent, Surrey and Sussex Air Ambulance Trust, Marden, Kent, UK; Pre-hospital Critical Care Services, The Central Denmark Region, Aarhus, Denmark; St George’s Hospital Medical School, University of London, London, UK; Emergency Department, St George’s Hospital, London, UK; Barts and The London School of Medicine and Dentistry, Garrod Building, Turner Street, Whitechapel, London, E1 2AD UK; The Institute of Pre-Hospital Care, 7-8 Philpot Lane, City of London, London, EC3M 8A UK; London’s Air Ambulance, Royal London Hospital, Whitechapel Road, London, E1 1BB UK; The Norwegian Air Ambulance Foundation, Holterveien 24, N-1441 Drøbak, Norway; Field of Pre-hospital Critical Care, University of Stavanger, Kjell Arholmsgate 41, N-4036 Stavanger, Norway; Dalhousie University Medical School, Halifax, Nova Scotia Canada; Trauma Nova Scotia, Halifax, Nova Scotia Canada; Department of Critical Care Medicine, Dalhousie University, Halifax, Nova Scotia Canada; Department of Critical Care Medicine, Dalhousie University, Halifax, Nova Scotia Canada; Division of General Surgery, Queen Elizabeth II Health Sciences Centre, Halifax, Nova Scotia Canada; Trauma Nova Scotia, Halifax, Nova Scotia Canada; ST3 Anaesthetist Barts Heart Centre, London, UK; FY2 Royal Victoria Hospital, Dundee, Scotland UK; Barts and the London School of Medicine and Dentistry, London, UK; Institute of Prehospital Care, London, UK; London’s Air Ambulance, London, UK; Department of Anaesthesia, King’s College Hospital, Denmark Hill, London, UK; Emergency Department, King’s College Hospital, Denmark Hill, London, UK; Department of Research and Development, Prehospital Emergency Medical Services, Central Denmark Region, 8200 Aarhus N, Denmark; Research Center for Emergency Medicine, Aarhus University Hospital, 8000 Aarhus C, Denmark; Department of Neuro Anesthesiology, Aarhus University Hospital, 8000 Aarhus C, Denmark; Barts and the London SMD, London, UK; Institute of Pre-Hospital Care, London’s Air Ambulance, London, UK; University of Cambridge, School of Clinical Medicine, Cambridge, UK; Essex + Herts Air Ambulance Trust, Colchester, UK; University of Cambridge, School of Clinical Medicine, Cambridge, UK; Essex + Herts Air Ambulance Trust, Colchester, UK; University of East Anglia, Norwich, UK; Essex + Herts Air Ambulance Trust, Colchester, UK; University of Leicester Medical School, Leicester, LE1 9HN UK; University of Cambridge Medical School, Cambridge, CB2 0SP UK; Essex and Hertfordshire Air Ambulance, Essex and Hertfordshire Air Ambulance Trust, Essex, UK; Trauma and Orthopaedics, Bradford Royal Infirmary, Bradford, West Yorkshire UK; General Surgery, Northern General Hospital, Sheffield, South Yorkshire UK; General Surgery, Scunthorpe General Infirmary, Scunthorpe, North Lincolnshire UK; North Bristol NHS Trust, Bristol, UK; Stewarts Law LLP, London, UK; University Hospital Southampton NHS Foundation Trust, Southampton, SO16 6YD UK; Department of Clinical Science and Education, Södersjukhuset, Karolinska Institutet, Stockholm, Sweden; Academic EMS, Stockholm, Sweden; St George’s University of London, London, UK; Major Trauma Unit, St Mary’s Hospital, Imperial College London, London, UK

## Abstract

I1: Trauma, Pre-hospital and Cardiac Arrest Care 2015

Pascale Avery, Leopold Salm, Flora Bird

A1: Retrospective evaluation of HEMS ‘Direct to CT’ protocol

Anja Hutchinson, Ashley Matthies, Anthony Hudson, Heather Jarman

A2 Rush hour – Crush hour: temporal relationship of cyclist vs. HGV trauma admissions. A single site observational study

Maria Bergman Nilsson, Tom Konig, Nigel Tai

A3 Semiprone position endotracheal intubation during continuous cardiopulmonary resuscitation in drowned children with regurgitation: a case report and experimental manikin study

Espen Fevang, Børge Hognestad, Håkon B. Abrahamsen

A4 An audit of CO2 A-a gradient in non-trauma patients receiving pre-hospital anaesthesia

Olivia V Cheetham, Matthew JC Thomas, Kieron D Rooney

A5 Can the use of c-spine immobilisation collars be avoided in non-trauma patients presenting to the Emergency Department?

Josephine Murray, Malcolm Tunnicliff

A6 Curriculum mapping in ED point of care simulation

Joseph W Collinson, Thomas Brown, Christopher Pritchett

A7 Point of care multidisciplinary trauma team simulation & participant satisfaction in a geographically remote trauma unit in Cornwall

Christopher SA Pritchett, Mark Jadav, Gareth Meredith, Jamie Plumb, Steve Harris, Roger Langford

A8 Conservative management of head injury inpatients - the challenge of simplifying injury management in a non-neurosurgical hospital

JG Hunter, A Sage, R Madden, O Flamank, B Broadbent, S Marsh, H Lewis, E Daniels, N Roberts

A9 Improving the care of traumatic brain injury at non-neurosurgical hospitals: Introducing a head injury pathway and single place of care is associated with significant improvements in neurological observation

JG Hunter, A Sage, R Madden, O Flamank, B Broadbent, S Marsh, H Lewis, E Daniels, N Lin, N Roberts

A10 The experience of inter-disciplinary students undertaking cardiac arrest moulage training

Samuel Bulford, Silas Houghton-Budd, Sam Pearson, Megan Clear-Hill

A11 Impact brain apnoea – nine cases

David J Menzies, James P Leonard, Conor Keogh, Ray Quinn, John D Hinds

A12 Time well spent? Improving the performance improvement programme in a busy Trauma Unit

N Roberts, D Ashton-Cleary, M Jadav

A14 Clinical significant and outcome of pulmonary contusions in patients with blunt chest trauma

Ismail Mahmood, Ayman El-Menyar, Basil Younis, Ahmed Khalid, Syed Nabir, Mohamed Nadeem Ahmed, Omer Al-Yahri, Hassan Al-Thani

A15 Plastics operative workload in major trauma centres: a national prospective survey

Katie Young, Susan A. Hendrickson, Georgina Phillips, Matthew D. Gardiner, Shehan Hettiaratchy

A16 A survey to assess the accuracy of estimating height by pre-hospital clinicians: can we reliably predict those most at risk of serious injury?

Alexandra Alice Crossland, Anthony Hudson

A17 An audit of the cause, outcome and adherence to treatment Standard Operating Procedure (SOP) for all traumatic cardiac arrests at a Helicopter Emergency Medical Service over a 12-month period

Nicholas C Brassington, Anthony Hudson, Emily McWhirter

A18 Should we “stay-and-play? A study of patient physiology in Norwegian Helicopter Emergency Services

Bjørn O Reid, Marius Rehn, Oddvar Uleberg, Andreas J Krüger

A19 Training in resuscitative thoracotomy: have we cracked it? A survey of higher Emergency Medicine trainees in London

Cara Jennings, Yasmin Kapadia, Duncan Bew

A20 London’s Air Ambulance (LAA): 25-years of drownings in an urban environment

Jenny Townsend, Tom P Hurst, Elizabeth A Foster

A21 Live patients in trauma simulation – more than just simulation on a shoestring?

Thomas B Brown, Joseph Collinson, Christopher Pritchett, Toby Slade

A22 Collecting core data in pre-hospital critical care using a consensus based template

Kristin Tønsager, Marius Rehn, Kjetil G.Ringdal, Andreas J.Krüger

A23 Prehospital interventions before and after implementation of a physician staffed helicopter

Rasmus Hesselfeldt, Sandra Wulffeld, Asger Sonne, Lars S. Rasmussen, Jacob Steinmetz

A24 Duration of ventilation following prehospital drug assisted intubation; a retrospective review

Thomas J Renninson, Nadine Thomson, Harvey Pynn, Timothy J Hooper

A25 Non-haemorrhagic shock in trauma: a novel guideline for management in ED

Anthony Hudson, Jacinta Dawson, Ashley Matthies

A26 Patient-tailored triage decisions by anaesthetist-staffed pre-hospital critical care teams

Morten Langfeldt Friberg, Leif Rognås

A27 Anatomical accuracy and appropriate sizing of pre-hospital thoracostomies

Jessica FG Wills, Anthony Hudson

A28 Pre-hospital management of mass casualty civilian shootings

Conor DA Turner, Marius Rehn

A30 The prevalence of alcohol-related trauma recidivism: a systematic review

James Nunn, Mete Erdogan, Robert S. Green

A31 Development of a hospital-wide program for simulation-based training in trauma care and management

Samuel Minor, Mete Erdogan, Kathy Hartlen, Robert S. Green

A32 Out of Hospital Cardiac Arrests (OOHCA); lessons from Hollywood

Ruth Bird, Rachael L. Grupping

A33 Mechanism of injury as a predictor of severity of injury in road traffic collisions: a literature review

Amelia M. Stacey, Marius Rehn, David J. Lockey

A34 Lessons to be learned from prehospital airway intervention documentation? Are airway intervention documentation templates as successful in-hospital as prehospitally?

S. Abiks, L. Cutler, K. Monaghan, A. Al-Rais, C. Hymers, R. Bloomer, Y. Kapadia

A35 Novel biomarkers in *pre*hospital management of *t*raumatic *b*rain *i*njury (the PreTBI study protocol)

Sophie-Charlott Seidenfaden, Ingunn S. Riddervold, Hans Kirkegaard, Niels Juul, Morten T. Bøtker

A36 Hospital outcomes of traumatic railway incidents: a seven-year observational retrospective study of a major trauma centre

Alice Gao, Zane Perkins; Gareth Grier, Alex Tzannes

A37 Does taking a third crew member affect the on-scene time of HEMS jobs?

Nathan Hudson-Peacock, Quentin Otto, Laurie Phillipson, Rik Thomas, Ainsley Heyworth

A38 Does pre-hospital rapid sequence induction affect on-scene time of HEMS jobs?

Quentin Otto, Nathan Hudson-Peacock, Laurie Phillipson, Ainsley Heyworth, Erica Ley

A39 Code red: shock index as a prehospital indicator of massive haemorrhage

Daniel Banner, Ainsley Heyworth, Erica Ley

A40 Air ambulance tasking: how accurate are our current methods?

Madeleine Benson, Nathan Hudson-Peacock, Tony Stone, Erica Ley, Louise Rousson, Ainsley Heyworth

A41 Modern trauma burden in a district general hospital

Beth A Lineham, Matthew J Lee, Martin Gough

A42 Establishing a legal service for major trauma patients in two UK major trauma centres

William H Seligman, Hannah E Thould, Andrew Dinsmore, Charlotte Tan, Julian Thompson, C Andy Eynon, David J Lockey

A43 Prehospital assessment and care of patients – a study of the use of guidelines when assessing head trauma

Rebecka M Rubenson Wahlin, Veronica Lindström, Sari Ponzer, Veronica Vicente

A44 An audit of pre-hospital blood pressure management resulting from head injury

Pamela Eligio, Anthony Hudson

A45 The surgical contribution of surface shading volumetric rendering techniques in rib fracture management

Robert Young, Dimitri Amiras, Ian Sinha

## I1 Trauma, Pre-hospital and Cardiac Arrest Care 2015

### Pascale Avery^1^, Leopold Salm^2^, Flora Bird^3^

#### ^1^The Royal Marsden Hospital, Chelsea, London, UK; ^2^The Royal London Hospital, London, UK; ^3^The Royal Free Hospital, London, UK

##### **Correspondence:** Pascale Avery (pascaleavery@live.co.uk) – The Royal Marsden Hospital, Chelsea, London, UK

**The 9**^**th**^**London Trauma Conference** (#LTC2015) and London Cardiac Arrest Symposium (#LCAS2015) built on the previous meetings with an emphasis on innovation, research, and enthusiasm for the medical care of major trauma, cardiac and critically ill patients. From the 8-11^th^ December 2015 delegates from over 20 countries attended The Royal Geographical Society for the four days of the conference. The opening two days of the conference focussed on current issues in major trauma, with air ambulance and pre-hospital critical care on day three, and the London cardiac arrest symposium returning as the fourth and final day. Concurrent breakaway sessions ran alongside the main conference including; trauma haemorrhage research, paediatric trauma, and masterclasses on cardiac ultrasound and resuscitation, thoracotomy, REBOA, and an introduction to ECLS and ECMO.

**The major trauma programme** consisted of two days of lectures, keynote lectures and short ‘quickfire’ sessions. **Professor Tim Coats** opened the conference by talking about the role of the highly performing trauma unit in trauma networks – outlining the problems of maintaining high levels of care in systems which increasingly bypass to major trauma centres but bring severely injured irregularly to trauma units. **Professor Kjetil Søreide** then addressed the topic of iatrogenesis in trauma, giving examples from different points in the patient pathway. The prevention of iatrogenesis is based on acceptance of it’s presence and then promoting prevention with a culture of safety, training and focus on the team approach. **Dr Matt Thomas** finished up by summarising the landscape of research in trauma over the previous year, as well as outlining what can be expected in the year ahead.

The following sessions approached key issues in neurotrauma, opened by a seasoned London Trauma Conference speaker **Mr Mark Wilson**. He spoke on current early neurological imaging, with mobile CT scanning already a reality in mainland Europe and the trialling of near infrared spectroscopy (NIRS) as a potential pre-hospital imaging modality. **Professor Geoffrey Raisman** followed with a fascinating talk on spinal cord regeneration, outlining how nerve regeneration to replace damaged portions has already been trialled with some success. He related a moving case where olfactory nerve fibres were used to repair spinal cord injury with one of the ultimate medical triumphs – making a paraplegic patient walk again. **Professor Andrew Maas** then lectured expertly on why he sees head injury as a silent epidemic with potentially life-changing consequences. **Dr Markus Skrifvars** closed the session with a sobering presentation on the link between alcohol consumption and the vast number of traumatic brain-injured patients that are intoxicated when they present.

Lunch was followed by **Professor Karim Brohi**, who delivered a talk on the early immune response to trauma and novel potential approaches to ameliorate this genomic storm. Other speakers in the afternoon included Professor Marc Turner delivering his vision for the trauma transfusion pack of 2025, and discussed whether stem cells may in the future become our source of blood for emergency transfusion. **Professor Susan Brundage** challenged the trauma myths of today, dismissing the Golden Hour as ancient history, with the platinum five minutes more relevant to 21^st^ century trauma. She concluded that our cultural understanding of context is crucial in understanding why myths occur and therefore how they can be dispelled.

Two overseas speakers addressed key issues in major incidents. **Professor Jeff Upperman** from Los Angeles spoke with passion, knowledge and experience on the challenging topic of paediatric penetrating trauma and the staggering gun violence statistics he faces in the US, with 355 mass shootings in less a year. The complexities of the gun lobby in the US are one of the many difficulties faced, and he emphasized the urgent need for action to reduce the current 3000 deaths in children from firearms injuries.

**Dr Ishay Ostfeld** presented the Israeli approach to mass casualty events. ‘Scoop and run’ has replaced the concept of ‘stay and play’, with triage occurring in hospital rather than in a pre-hospital setting. They aim to have all severely injured patients evacuated within 30 minutes and most receive operative intervention within 90 minutes.

The day two morning session heard **Dr Conor Deasy** speak about trauma team performance, highlighting the advantages that can be gained from ‘in situ’ simulation and the use of video. **Professors Wolfgang Voelckel** and **Simon Carley** spoke about the fascinating complexities of decision-making and clinical judgement in the resus room. The latter encouraged delegates to analyse and utilise their feedback, reflect on case notes and be disciplined in learning about ones thinking processes.

**Mr Ross F**isher began a paediatric themed series of talks by arguing the case for specialised paediatric trauma units. This was supported by **Dr Natalie May** who reiterated the challenges faced when treating these patients. An informative summary of how to approach paediatric imaging was provided by Radiologist **Dr Caren Landes**, who referred to the revised NICE guidelines for the indications for CT head and c-spine imaging. A personal and reflective account by **Kirsti Soanes** addressed how best to manage the parents of a child during paediatric resuscitation.

**Dr Jeff Upperman** provided a dynamic introduction to the afternoon session on major incidents. He spoke about preparedness, the importance of recovery and resilience, and argued that planning for children tends to be inadequate. The Peter Baskett memorial lecture was given by **Professor Pierre Carli** who gave an insightful and impressive account of France’s response to the recent terrorist attacks in Paris. This was followed by an international panel discussion with representatives from the US, Norway, Israel and France. They highlighted the importance of lessons that can be learnt from the experiences of others, and discussed topics that included triage systems, the proximity of EM personnel to the ‘hot zone’ and how best to recognise when a system is overwhelmed.

**Professor Anders Oldner** then spoke about the post resuscitation phase of care in trauma patients and the significant challenge posed by post injury sepsis. **Mr Jan Jansen** provided a surgeon’s insight into penetrating and blunt cerebrovascular neck injuries. He advocated the strength of CT angiogram in detecting injuries and the use of selective non-operative management when indicated. The final session of the afternoon consisted of ‘quickfire’ ten minute talks that addressed the current barriers to trauma care, when heparins should be used after trauma, and the merits of tranexamic acid in bleeding trauma patients.

**The air ambulance and pre-hospital care day** was opened by **Gen. Sec. Professor Hans Morten Lossius** of the Norwegian Air Ambulance, and focused on air ambulance and pre-hospital care. Themes of new technology and techniques ran throughout, including ECMO, REBOA, video laryngoscopy, and point of care testing.

**Professor Pierre Carli** head of SAMU in Paris gave an illuminating lecture on use of pre-hospital ECMO for refractory cardiac arrest. With considerable experience in Paris, he reported good feasibility, and data showing increasing survival rates in pre-hospital extra-corporeal life support with changes in inclusion criteria, training, and limitation of adrenaline. Furthermore, there has been improved organ donation following therapeutic ECLS.

**Dr Thomas Linder** spoke about the new ERC guidelines, reinforcing that chest compressions are still the mainstay of effective CPR. The question ‘Rapid response cars; more dangerous than the aircraft?’ was answered by **Dr Marius Rehn**. Lessons were drawn from motorsport and aviation, with an overview of safety techniques used by the rapid response cars at London’s Air Ambulance, including car trackers, cameras, and checklists.

**Associate Professor Andrew Pearce**’s lecture ‘The Golden Day: making the most of long distance critical care’ demonstrated the challenges of Australian geography. He provided three important messages: you are never alone, it is a system approach, and communication is vital. ‘An agitated patient could be a dying patient trying really hard to stay alive’ was the message from **Mr Andy Thurgood**. He reminded us we must still apply a checklist to the agitated patient, and be aware that agitation is emotive and can affect rescuer performance.

Recent experiences of setting up national retrieval services were recounted for Demark and Wales. **Dr Leif Rognas** developed a government funded anaesthetic led service in Denmark, covering the entire population, working 24/7. He reported on 13 months of service and challenges including the integration of service into the entire EMS, and standardising joint SOPs. **Dr Rhys Thomas** has led development of a new a consultant delivered seven day a week service in Wales. He emphasised the importance of data collection for service evaluation and development.

**Dr Per Kristian Hyldmo** addressed the controversial issue of spinal immobilization during intubation, followed by **Mr. Tom Judge** who shone a spotlight onto the financial implications of the US air ambulance services. He talked about an abundance of helicopter supply, driven by demand. Commercial pressures rather than clinical need may drive competition to get patients on board the helicopter.

**Professor Sir Simon Wessely** gave an insightful keynote lecture on mental health, concluding that people will withstand enormous hardship and show resilience, if they feel there is a meaning or purpose to doing so. He differentiated transient hardship and genuine mental health problems with long term consequences – only found in a minority. Resources should be directed to the minority who are not getting better.

‘Pre-hospital Sepsis’, and ‘Point of care testing in pre-hospital haemorrhage’ were discussed by **Professor Kai Zacharowski**. He discussed The UK Sepsis Trust screening tool for pre-hospital identification of sepsis, and importance of ‘sepsis 6’ management. Point of care testing enabled significantly faster goal directed therapy.

**Dr Julian Thompson** highlighted the challenges of implementing pre-hospital CRM and SOPs in hospital, concluding these procedures are highly applicable to high risk situations, requiring small well governed teams at the points of greatest need. A year after introduction of the procedure seven cases of pre-hospital resuscitative endovascular balloon occlusion of the aortaREBOA was reviewed by **Dr Samy Sadek** from London who concluded that training well with tight clinical governance and careful patient selection produces good results. **Dr Matt Thomas** spoke on use of PALM in polytrauma patients with reduced GCS and on-going airway obstruction, and the danger of ‘no plan B’. **Mr Tom Judge** presented in support of using video laryngoscopy in pre-hospital care.

**The London Cardiac Arrest Symposium** was focused on insight into and innovation around cardiac arrest care.

**Professor Charles Deakin** opened the day by discussing the controversial topic of epinephrine in cardiac arrest. He suggested that the evidence for neurologically intact survival points to benefit from early epinephrine, and worsening the outcome if given late. A systematic review and meta-analysis in 2014 reported that overall epinephrine does not improve survival. The results of the Paramedic 2 prospective RCT of 8000 patients, epinephrine vs placebo are awaited. **Mr Ken Spearpoint** then spoke on psychological outcomes in recovery from cardiac arrest.

‘The Failing Heart Post ROSC’ was a topical talk from **Dr Simon Finney** covering the complex network of interactions in myocardial dysfunction, and modalities for measuring cardiac output. **Professor Philip MacCarthy** addressed the roles of ‘Cath Labs in Cardiac Arrest’. The ECG is unreliable in predicting vessel occlusion. He presented evidence suggesting incidence of coronary artery disease in NSTEMI is 52 %, and STEMI 93 %; noting in the PROCAT study 26 % of NSTEMI patients had a culprit lesion. He asked ‘Should be taking all to the Cath Lab?’, and concluded with the need for vigorous implementation of effective patient pathways.

**Prof James Manning** spoke passionately about emerging endovascular and extracorporeal therapies in resuscitation, describing an ‘interventional toolkit tailored to the needs of individual patients’. **Dr Conor Deasy** described measures used in Ireland to improve outcomes in rural cardiac arrest, including community based education, improving access to defibrillators, and drone cameras carrying AEDs.

An ERC guideline update was given by **Professor Charles Deakin**. He emphasised the importance of early quality bystander CPR/AED, stepwise airway management, and mandatory waveform capnography. Evidence from 3 RCTs (n = 7582) showed no clear advantage for mechanical compression device vs. manual chest compression, but concluded it is a reasonable alternative where a sustained high quality manual chest compression is impractical or unsafe.

**Professor Simon Redwood** updated on automated CPR and its role in today’s clinical practice. This was followed by the **Professor Tim Harris** on the topic of monitoring the acutely unwell heart post cardiac arrest. He identified stroke volume as the measurable current variable of choice for assessing fluid requirements. However, he noted that a clearer understanding of the microcirculation and routine ways to measure it would be a better predictor of not only mortality, but length of hospital stay. He argued that we should not strive for the top of the Starling curve but consider tissue perfusion to be the central principle guiding appropriate fluid therapy.

The final afternoon session of the cardiac arrest symposium commenced with **Mark Whitbread** defining clear differences between ventricular fibrillating states. Although there has been an increased survival, this pathology still carries a very high mortality rate where defibrillation is delayed and warrants urgent transportation to a cardiac centre for ongoing management**. Dr Rob Morrison** addressed the topical subject of pitfalls to best practice in considering Do Not Attempt Resuscitation orders, where the key lies in clear communication. Closing the 2015 cardiac arrest symposium, **Dr Marius Rehn** delivered a sobering presentation on cardiac arrest through hanging, its considerable associated mortality, and the essential need to assess for soft tissue injuries with this mechanism.

**Research and prizes:** 45 abstracts were accepted for poster and oral presentation at the London Trauma Conference 2015. Eight authors faced the ‘Stand up science’ session to enthusiastically present their work to an evening audience, with Dr David Menzies being awarded the oral presentation prize for his presentation on impact brain apnoea. The winner of the poster prize was Dr William Seligman and colleagues for their work on the introduction of legal trauma services to major trauma centres.

## A1 Retrospective evaluation of HEMS ‘Direct to CT’ protocol

### Anja Hutchinson, Ashley Matthies, Anthony Hudson, Heather Jarman

#### Emergency Department, St. Georges University Hospital, London, UK

##### **Correspondence:** Anja Hutchinson (m1302423@sgul.ac.uk) – Emergency Department, St. Georges University Hospital, London, UK

Background

Early whole body CT has an established survival benefit for trauma patients [1]. Many trauma patients arriving at St Georges Emergency Department (ED) arrive with Helicopter Emergency Medical Services (HEMS) having had a thorough primary survey and basic early interventions. To avoid delay in CT time due to repetition of primary survey in the ED, haemodynamically stable patients arriving with HEMS enter a ‘Direct to CT’ protocol which this study evaluated for safety and efficacy.

Methods

This was a retrospective evaluation of patients arriving at St George’s Hospital accompanied by HEMS over a 6 month period from April 2014. Data was obtained from EPR, PACS, EDM, patients notes and clinicians. Adverse events were complications during scanning or missed time critical injuries. Time to CT was measured from booking to the time CT was performed.

Results

Of the 222 patients admitted 62 went directly to CT, 73 did not go directly to CT and 87 could not be determined. No adverse events were recorded and average time to CT in the protocol was 19 mins, compared to 28 mins average for all patients admitted. Primary identifiable reasons for exclusion were age (paediatrics were excluded to ensure radiation exposure was warranted) and due to haemodynamic instability. Male to female ratio was 3:1 indicating representative population of trauma patients.

Conclusion

The study found HEMS ‘Direct to CT’ Protocol to be both safe and effective. Average time to CT was reduced by 9 mins through the protocol and no adverse events were recorded. The authors would recommend this protocol to other EDs and recommend expanding the protocol to include other seriously injured patients arriving without the assistance of HEMS.

Reference

1. Huber-Wagner S, Lefering R, Qvick LM, et al. Effect of whole-body CT during trauma resuscitation on survival: a retrospective, multicentre study. *The Lancet.* 2009; 373:1455-1461.

## A2 Rush hour – Crush hour: temporal relationship of cyclist vs. HGV trauma admissions. A single site observational study

### Maria Bergman Nilsson^1^, Tom Konig^2^, Nigel Tai^2^

#### ^1^Queen Mary University of London, Barts and The London School of Medicine and Dentistry, Whitechapel, London, UK; ^2^Dept of Trauma Surgery, Barts Health NHS Trust, Whitechapel High St, London, E1 1BB, UK

##### **Correspondence:** Maria Bergman Nilsson (ha11321@qmul.ac.uk) – Queen Mary University of London, Barts and The London School of Medicine and Dentistry, Whitechapel, London, UK

**Introduction**

London has seen a significant increase in cycle usage in light of Team GB successes post London Olympics 2012. Increased cycle journeys heralded a drive for cycle lanes, but the cost justification has been questioned. A global city attracts highly skilled, health conscious workers; cycling and its associated risks have become a planning issue. Recent highly publicised deaths involving heavy goods vehicles (HGVs) begged questions of possible safety interventions and potential restrictions. In order to identify any, we looked to the timing of cycle related trauma admissions.

Methods

Retrospective database analysis of cyclist admissions over a 10-year period was conducted and reviewed in terms of admission time and type of vehicle. Reports using the word “truck”, “lorry” or “HGV” were classified similarly. These were compared with timings of admissions of casualties associated with cars.

Results

Admission time of cyclists involved in collision with HGVs showed significant differences compared to collisions with cars. Close to 50 % of all HGV casualties took place over the time period recognised as a typical morning rush hours period. A further peak was seen at or around midday. These peaks coincide with peak time for traffic and goods delivery.

Conclusion

Strategic changes to improve road safety were suggested in the 2012 roads task force, specifically the restriction of the time HGVs operate. This was achieved during the London Olympics when HGVs were restricted to “out of hours” [1]. Improvement in cyclist mortality during this time is unclear. Paris has operated a similar approach restricting larger goods vehicles to the period 1930-0730. In light of our findings and existing evidence, further study should be undertaken across London to determine if local results translate pan-London and whether there should be a change in the times of access of HGVs, or if not, the provision of trauma services and the allocation of resources.

**Reference**

1. Transport for London, Mayor of London. Safe Streets for London The Road Safety Action Plan for London 2020. 2014;88. Available from: http://tfl.gov.uk/corporate/safety-and-security/road-safety/safe-streets-for-london?intcmp=6624

## A3 Semiprone position endotracheal intubation during continuous cardiopulmonary resuscitation in drowned children with regurgitation: a case report and experimental manikin study

### Espen Fevang^1,2^, Børge Hognestad^1^, Håkon B. Abrahamsen^1,2^

#### ^1^Department of Research and Development, Norwegian Air Ambulance Foundation, Drøbak, Norway; ^2^Department of Anaesthesiology and Intensive Care, Stavanger University Hospital, Stavanger, Norway

##### **Correspondence:** Espen Fevang (espen.fevang@norskluftambulanse.no) – Department of Anaesthesiology and Intensive Care, Stavanger University Hospital, Stavanger, Norway

Background: Drowning continues to be one of the leading causes of accidental deaths in children worldwide. Prompt treatment of hypoxemia with positive pressure ventilation improves survival, but regurgitation of pulmonary and gastric contents complicates airway management. Hands-off time during cardiopulmonary resuscitation **(**CPR) can be detrimental to the patient [1].

In August 2010 a car containing two children aged three and six went off a pier into seawater. When rescued both had confirmed asystole and dilated unreactive pupils, with a submersion time of approximately 15 minutes. Within three minutes after rescuing both had been successfully endotracheally intubated in the semiprone position during continuous CPR. This allowed for the passage of gastric and pulmonary contents throughout the procedure. Both children had return of spontaneous circulation within 30 minutes and were discharged from hospital without sequelae two and three weeks after the accident.

Our objective was to re-assess the feasibility of endotracheal intubation **(**ETI) during continuous CPR in the semiprone position compared to a standard supine position in a simulated drowned child.

Method: We conducted a manikin experiment where five experienced providers performed ETI on a SimJunior® with simulated regurgitation during continuous CPR in the supine and semiprone position.

Results: During simulated regurgitation and continuous CPR, all participants managed visually confirmed ETI within 15 seconds with a mean time to intubation of 11.7 seconds in the semiprone position. No participants managed visual confirmation of ETI in the supine position. Three of five managed to blindly intubate the simulation dummy through the regurgitation debris in the supine position, with a mean attempt time of 16.9 seconds.

Conclusion: In a simulation-based model, we have shown that ETI in the semiprone position during continuous CPR can be a fast and reliable backup option when airway management is complicated by regurgitation in children with submersion injuries.

Reference

1. Szpilman D, Bierens JJ, Handley AJ, Orlowski JP. Drowning. *N Eng J Med*. 2012; 366 (22); 2102-2110.

## A4 An audit of CO2 A-a gradient in non-trauma patients receiving pre-hospital anaesthesia

### Olivia V Cheetham^1^, Matthew JC Thomas^2,3^, Kieron D Rooney^2,3^

#### ^1^University Hospitals Bristol, Bristol, UK; ^2^University Hospitals Bristol, Bristol, UK; ^3^Great Western Air Ambulance, South West Ambulance Service Trust, Exeter, UK

##### **Correspondence:** Olivia V Cheetham (oliviacheetham@googlemail.com) – University Hospitals Bristol, Bristol, UK

**Background**

Sedation and mechanical ventilation are often indicated in patients who achieve a return of spontaneous circulation (ROSC) following an out of hospital cardiac arrest (OHCA). An appropriately trained critical care team can deliver this in the pre-hospital environment.

Both hypercapnia and hypocapnia correlate with poor neurological outcome [1]. In the absence of invasive blood gas analysis, end-tidal CO2 (EtCO2) can be used to estimate arterial carbon dioxide tension (PaCO2). In healthy individuals, the difference between PaCO2 and EtCO2 (the A-a gradient) is negligible [2], but reduced cardiorespiratory function can cause ventilation and perfusion (VQ) mismatching and larger gradients. The American Heart Association (AHA) guidelines for post- cardiac arrest care recommend targeting EtCO2 from 4.67-5.33 kPa and PaCO2 from 5.33-6.0 kPa [3]. We sought to identify whether a clinically significant A-a gradient exists in this patient group and whether the recommended PaCO2 is achieved by altering ventilation strategies using EtCO2 levels.

Method

Twenty-seven non-trauma patients, of which 22 were OHCA, underwent pre-hospital rapid sequence induction of anaesthesia (RSI) and were conveyed to the University Hospitals Bristol, a tertiary cardiac arrest centre, between April 2013 and May 2015. Their case notes were reviewed retrospectively and paired EtCO2 and PaCO2 values on arrival to hospital were recorded. Each patient had an A-a gradient calculated.

Results

Mean EtCO2 on admission was 4.94kPa (95 % C.I. 4.48-5.4) and mean PaCO2 was 7.12kPa (95 % C.I. 6.46-7.78). PaCO2 values were higher than EtCO2 on average by 2.18kPa (A-a gradient).

Conclusion

EtCO2 targets are being met by the pre-hospital critical care team but patients are hypercapnic upon hospital arrival. There is a large A-a gradient due to VQ mismatching, possibly due to increased dead space. This preliminary data suggests we should be targeting a lower EtCO2 in post-ROSC and peri-arrest patients undergoing RSI to achieve normocapnia and optimise neurological outcome.

**References**

1. Roberts BW, Kilgannon JH, Chansky ME, Mittal N, Wooden J and Trzeciak S. Association Between Postresuscitation Partial Pressure of Arterial Carbon Dioxide and Neurological Outcome in Patients With Post-Cardiac Arrest Syndrome. *Circulation.* 2013; 127; 2107–2113.

2. Oridion Medical. Understanding the Arterial to End Tidal CO_2_ Gradient; P(a-et)CO_2_. 2009 [online] http://www.procamed.ch/pdf/etco2_gradient.pdf [Accessed November 4, 2015]

3. Peberdy MA, Callaway CW, Neumar RW, Geocadin RG, Zimmerman JL, Donnino M, Gabrielli A, Silvers SM, Zaritsky AL, Merchant R, Vanden Hoek TL and Kronick SL. 2010 American Heart Association Guidelines for Cardiopulmonary Resuscitation and Emergency Cardiovascular Care. Part 9: post-cardiac arrest care. *Circulation.* 2010; 122; S768–S786.

## A5 Can the use of c-spine immobilisation collars be avoided in non-trauma patients presenting to the Emergency Department?

### Josephine Murray, Malcolm Tunnicliff

#### Emergency Department, King’s College Hospital, London, UK

##### **Correspondence:** Josephine Murray (Josie.murray@doctors.org.uk) – Emergency Department, King’s College Hospital, London, UK

**Background**

Current resuscitation guidance such as Advanced Trauma Life Support (ATLS) advises that all trauma patients with potential cervical spine (c-spine) injury should be immobilised in a rigid collar and blocks until significant c-spine injury has been excluded. However, increasing evidence suggests that the use of these collars may actually be more harmful then beneficial in patients with low risk of cervical spine injury [1]. Potential harm from rigid collars includes increased neck movements, aspiration and raised intra-cranial pressure [2,3].

This study aims to utilise a selection criteria to identify low risk trauma patients and assess the frequency of c-spine pathology amongst these patients. The results of this study will help determine whether new guidance will be implemented to minimise the unnecessary use of collars in these patients.

**Method**

All c-spine x-rays taken over a 7-month period (22/9/14 – 30/4/15) were identified and the results recorded for patients deemed low risk. A selection criterion for low risk trauma patients was utilised which included GCS 15/15, no distracting injuries, non-major trauma and the absence of a dangerous mechanism.

**Results**

A total of 270 x-rays were identified, of which 73 patients qualified for this study. Acute c-spine pathology was identified in 1 of these patient. The x-ray from this patient identified a possible pseudo-subluxation after which the patient was discharged without follow-up by the neurosurgical team.

**Conclusion**

The results of this study suggest that there is a low frequency of c-spine pathology amongst non-major trauma patients presenting with a GCS 15/15 without distracting injuries, and that these patients can be identified using the proposed selection criteria. The isolated identified pathology in this study was a stable injury and managed with conservative treatment only. The results also support evidence found in a previous study that suggests collars in this subgroup of patients may outweigh the benefits^1^, and recommends the use of blocks only. Our findings support the introduction of a new protocol at the Accident and Emergency Department at King’s College Hospital to identify these patients and use only blocks for their immobilisation.

**References**

1. Holla M: Value of a rigid collar in addition to head blocks: a proof of principle study. *Emergency Medicine Journal*. 2012; 29: 104-107.

2. Ho AM, Fung KY, Joynt GM, Karmaker MK, Peng Z: Rigid cervical collar and intracranial pressure of patients with severe head injury. *Journal of Trauma, Injury Infection and Critical Care.* 2002; 53(6): 1185-1186.

3. Papadopoulos MC, Chakraborty A, Bell AB. Exacerbating cervical spine injury by applying a hard collar. *British Medical Journal*. 1999; 319(7203): 171-172.

## A6 Curriculum mapping in ED point of care simulation

### Joseph W Collinson, Thomas Brown, Christopher Pritchett

#### Emergency Department, Royal Cornwall Hospital, Truro, UK

##### **Correspondence:** Joseph W Collinson (joseph.collinson110@gmail.com) – Emergency Department, Royal Cornwall Hospital, Truro, UK

**Background**

Simulation is an effective tool to teach the core competencies of the Emergency Medicine (EM) curriculum [1]. Previous studies have shown that curriculum maps optimise teaching programmes in medical education and result in high student satisfaction [2]. We set out to design and implement an Emergency Department (ED) simulation programme with an associated curriculum map to cover the major presentations listed in the Royal College of Emergency Medicine curriculum. We present our preliminary findings using this approach.

**Methods**

We designed and implemented a 6 month rolling ED simulation programme to cover the major presentations listed in the Emergency Medicine curriculum – 6 critical illness, 6 paediatric, and 6 major trauma. Each week a multi-disciplinary point of care simulation was delivered in the Emergency Department of the Royal Cornwall Hospital, Truro. The simulations were led by an ED consultant, with middle grades, junior doctors and nursing staff participating. A curriculum map was designed for each session outlining the specific EM curriculum points covered in that session. Trainee perception of the relevance of simulation to their training and to their curriculum was assessed using a ten point visual analogue scale before and after the introduction of a curriculum map.

**Results**

Trainees (n = 11) felt that a curriculum map would be very useful (mean score 9/10) to aid their learning. While trainees felt that simulation was relevant to their curriculum generally (mean score 9.2/10), they did not know specifically what learning points were in the curriculum and how these related to simulation (mean score 4.3/10). Following the design of a curriculum map this score increased to 9/10.

**Conclusions**

Simulation training in the ED is an effective tool for improving trainee performance [1]. We have shown that comprehensive curriculum mapping in this context is valued by trainees, and improves trainee familiarity with their curriculum.

References

1. Ten Eyck RP Simulation in emergency medicine*. Paediatric Emergency Care.* 2011; 27(4):333-341.

2. Wong R, Roberts J. Real time curriculum maps for internal medicine residency. *BMC Medical Education* 2007; 7: 42.

## A7 Point of care multidisciplinary trauma team simulation & participant satisfaction in a geographically remote trauma unit in Cornwall

### Christopher SA Pritchett, Mark Jadav, Gareth Meredith, Jamie Plumb, Steve Harris, Roger Langford

#### Simulation Department, Royal Cornwall Hospital, Truro, UK

##### **Correspondence:** Christopher SA Pritchett (Christopher.pritchett@doctors.org.uk) – Simulation Department, Royal Cornwall Hospital, Truro, UK

Background

Effective coordination and rapid assessment by a multi-disciplinary trauma team (MDTT) has been shown to reduce mortality (1). Difficulties are exacerbated when MDTTs rarely work together, are assembled ad-hoc and are exposed to infrequent major trauma incidents.

Simulation training has been proposed as a safe and effective tool, supported by adult learning theory. (2,3)

The Royal Cornwall Hospital is a geographically remote district hospital and Trauma Unit. Patients arriving in the resuscitation room may have a prolonged primary and secondary transfer time. Trauma simulations have been run for the last 4 years. In 2013 these became ‘point of care’: run monthly in real time in the resuscitation room by staff working regularly there. In this baseline service evaluation we sought to assess domain 1 ‘Reaction’ of the Kirkpatrick educational model (2).

Methods

Data was collected from trauma simulation run at point of care from April 2013 to October 2015.

Data collected included total number of scenarios, job role of participants and anonymised feedback following a formal debrief of the scenario by a trained facilitator.

Results

Over 31 months, 21 MDTT scenarios were run. 201 medical professionals & students were trained (115 Doctors, 28 nurses & 37 students). Session cancelation rate was 32 %.

Participant’s satisfaction: Lickert scales were used to assess participant views on clinical realism, influence on their clinical ability & educational value of the scenario. Mean scores ranged from 7.7-10 on a 10-point scale. Nurses rated the sessions higher than doctors in every domain.

Conclusions

As a remote trauma unit with prolonged primary & secondary transfer times, maximising performance of the trauma team through simulation is an effective educational tool.

We have installed an effective, regular MDTT simulation with high participant satisfaction scores. Future goals must include increasing staff availability for simulation & establishing then tackling causes for cancelation.

References

1. Georgiou A, Lockey DJ. The performance and assessment of hospital trauma teams. *Scand J Trauma Resusc Emerg Med*. 2010; 18: 66

2. Gjerraa K, Møller TP and Østergaard D. Efficacy of simulation-based trauma team training of non-technical skills. A systematic review. *Acta Anaesthesiol Scand.* 2014; 58: 775-787.

3. Mercer S, Park C and Tarmey NT. Human factors in complex trauma. *BJA Education*. 2015; 15(5): 231-236.

## A8 Conservative management of head injury inpatients - the challenge of simplifying injury management in a non-neurosurgical hospital

### JG Hunter^1^, A Sage^2^, R Madden^1^, O Flamank^2^, B Broadbent^2^, S Marsh^2^, H Lewis^2^, E Daniels^2^, N Roberts^1^

#### ^1^Emergency Department, Royal Cornwall Hospital (RCH), Truro, UK; ^2^Peninsula School of Medical and Dentistry, Royal Cornwall Hospital (RCH), Truro, UK

##### **Correspondence:** JG Hunter (jghunter@doctors.org.uk) – Emergency Department, Royal Cornwall Hospital (RCH), Truro, UK

**Background**

Traumatic brain injury has an incidence of 9 per 100,000 per year [1]. Conservative management at non-neurosurgical centres is common but challenging given lack of appropriately trained inpatient teams. Initial audit at a non-neurosurgical district general hospital (DGH) between 2012-14 found variable destinations of care and poor observation and escalation. Post admission ward was often determined by co-existing injuries and co-morbidities. In August 2014 a simplified head injury pathway was introduced where all inpatients were to be managed on the Clinical Decision Unit (CDU) of the Emergency Department (ED) for the first 24 hours prior to onward referral to a neurology ward if still symptomatic. This re-audit assesses compliance with this new policy.

**Methods**

Notes were retrospectively reviewed for all adults with traumatic intracranial haemorrhage admitted via ED between September 2014 and February 2015. Destination of care beyond ED was recorded along with reasons for deviation from this pathway.

**Results**

45 patients were included. 58 % (n = 26) were initially admitted to CDU, 36 % (n = 16) admitted to medical admissions, 4 % (n = 2) direct to neurology and 2 % (n = 1) to critical care. Of those admitted to CDU, 50 % (n = 13) were safely discharged, 15 % (n = 4) were admitted to neurology, with 35 % (n = 9) transferred to outlying medical and surgical wards. 7 % (n = 3) were urgently transferred to a neurosurgical centre after admission. Reasons for deviation included co-morbidities requiring specific inpatient management, bed unavailability and poor awareness of the new policy.

**Conclusions**

This simplified traumatic head injury management pathway through CDU was designed to provide referral clarity and build clinical management expertise. Implementing this pathway has been challenging, particularly in the context of increasing bed pressure and an ageing co-morbid population. Further research is needed to assess the impact of this on care quality and outcomes.

Reference

1. Teasdale GM. Head injury. J Neurol Neurosurg Psychiatry. 1995;58(5):526- 539.

## A9 Improving the care of traumatic brain injury at non-neurosurgical hospitals: Introducing a head injury pathway and single place of care is associated with significant improvements in neurological observation

### JG Hunter^1^, A Sage^2^, R Madden^1^, O Flamank^2^, B Broadbent^2^, S Marsh^2^, H Lewis^2^, E Daniels^2^, N Lin^3^, N Roberts^1^

#### ^1^Emergency Department, Royal Cornwall Hospital (RCH), Truro, UK; ^2^Peninsula School of Medical and Dentistry, Royal Cornwall Hospital (RCH), Truro, UK; ^3^Department of Mathematics and Information Sciences, Northumbria University, Newcastle upon Tyne, UK

##### **Correspondence:** JG Hunter (jghunter@doctors.org.uk) – Emergency Department, Royal Cornwall Hospital (RCH), Truro, UK

**Background**

Traumatic brain injury (TBI) is a significant cause of adult mortality and disability in the United Kingdom. Conservative management of these injuries at non-neurosurgical centres is common, reliant on close observation and liaison with specialists. Initial audit at a non-neurosurgical district general hospital (DGH) between 2012-14 found multiple destinations of care within the hospital and poor levels of neurological observation. This relook audit assesses the impact of introducing a simplified TBI management pathway with stable patients observed for the first 24 hours on the Clinical Decision Unit (CDU).

**Methods**

Retrospective audit of all adult head injuries admitted via the Emergency Department (ED) at a DGH between September 2014-February 2015 with complete medical notes available for analysis. Charts were scored against national guidance for head injury observation.^1^ Statistical analysis utilized the ordered logit model for ordered categorical data.

**Results**

The initial audit analysed 87 patients and the relook included 37 patients. Initially 8 % of patients received correct levels of observations until GCS = 15. Post intervention this improved to 28 %. Observations for the first 2 hours improved, with those getting 0-1 sets of observations dropping from 55 % to 30 %, 2-3 sets improving from 30 % to 54 %, and 4 sets improving from 15 % to 16 % (p = 0.037). Observation levels pre- and post- intervention followed a similar trend for the subsequent 4 hours of observation (p = 0.984). Appropriate escalation following deterioration improved by 16 % when admitted to CDU.

**Conclusions**

A change in policy to managing all TBI patients for the first 24 hours in CDU rather than on various medical or surgical wards significantly improved the quality of their neurological observation. There is still scope for improvement but this is encouraging. This principle could be applied at other non-neurosurgical centres to improve the care of this potentially unstable group of patients.

**Reference**

1. Head injury (2014) NICE guideline CG176.

## A10 The experience of inter-disciplinary students undertaking cardiac arrest moulage training

### Samuel Bulford^1^, Silas Houghton-Budd^2^, Sam Pearson^2^, Megan Clear-Hill^1^

#### ^1^Medical School, University College London, London, UK; ^2^Ashford make-ready station, South East Coast Ambulance Service, Ashford, UK

##### **Correspondence:** Samuel Bulford (Samuel.bulford.09@ucl.ac.uk) – Medical School, University College London, London, UK

**Background**

Globally, healthcare students highlight the educational benefit of spending time with pre-hospital clinicians [1,2]. In the UK there is considerable medical student interest in this increasingly emerging field. Equally, there is increasing educationalist interest in ‘medutainment’ and observed simulation [3]. We wanted to prove that inter-disciplinary, simulation ‘medutainment’ in pre-hospital care benefits a training clinician.

**Method**

Medical and paramedic students planned and delivered simulation teaching on cardiac arrest management. Inter-disciplinary student teams worked in real-time high fidelity moulages wherein they managed a cardiac arrest patient out of hospital before transferring to, and continuing the care, in hospital. All participants adopted both pre- and in-hospital roles. When not actively participating, participants were able to observe via live video link. Participants completed a pre- and post-event quantitative and free space questionnaire to evaluate the efficacy of this novel educational intervention.

**Results**

The event was exceptionally well received. Students learnt the medical management of cardiac arrest, but also learnt about human factors and crew resource management. More broadly, students learnt about the practices, capabilities and limitations of their colleagues’ profession, and the different considerations of the in- and out-of hospital clinician. Students remarked that this shared understanding will help them better appreciate and respect their colleagues in future. Student also commented that the event has potential to change their practice given the broader knowledge of a patient’s care and journey. Finally, students remarked that collaborative learning helps erode the ‘them and us’ attitude sometimes seen between different clinicians.

**Discussion**

This student devised and led event demonstrates that there is tremendous benefit in engaging inter-disciplinary students from an early stage of training. It demonstrates that students are aware of cultural differences between professions - and equally some prejudice. Pleasingly, though, it also demonstrates that this collaborative learning has the potential to lead to greater, more holistic, collaborative practice.

References

1. Melby, V. The adrenaline rush: nursing students’ experiences with the Northern Ireland Ambulance Service. *Journal of Advanced Nursing* 2001; 34 (6): 727-736.

2. Dickinson, WW. Pre-hospital trauma management. *Accident and Emergency Nursing*. 1994; 2: 2–6.

3. Jarvin, L. Edutainment, games, and the future of education in a digital world. *New Dir Child Adolesc Dev* 2015; 147: 33-40.

## A11 Impact brain apnoea – nine cases

### David J Menzies^1,2^, James P Leonard^1^, Conor Keogh^1^, Ray Quinn^1^, John D Hinds^1^

#### ^1^Motorcycling Ireland Medical Team, Dublin, Ireland; ^2^Emergency Department St Vincent’s University Hospital, Dublin, Ireland

##### **Correspondence:** David J Menzies (d.menzies@svuh.ie) – Emergency Department St Vincent’s University Hospital, Dublin, Ireland

**Background:** Impact brain apnoea (IBA) is an under reported contributor to mortality and morbidity in head injured patients. It is encountered only when there is a medical response within the first few minutes of a head injury, with the result that there is little published on it in the medical literature. The Motorcycling Ireland Medical Team encounters impact apnoea with relative frequency and we describe nine consecutive cases.

**Method:** Patient Care Reports from race events in the Republic of Ireland attended by our medical team between 2009 and 2015 were searched to identify patients with a head injury who were apnoeic on first medical assessment. Treatment, injuries and outcome were documented.

**Results:** We report a case series of nine patients with clinical signs consistent with IBA.

All were victims of high-speed motor collisions and all were apnoeic and cyanotic at scene. Three patients were treated with basic airway manouveurs alone, six patients underwent Rapid Sequence Intubation (RSI) at scene.

Each patient made a full neurological recovery with mild - moderate underlying traumatic brain injuries. We did not witness significant haemodynamic compromise in this patient group as has been reported elsewhere.

**Conclusions:** IBA is an under-reported phenomenon, only encountered when there is an ultra rapid medical response to a head injury. In spite of high-risk mechanisms and other significant injuries, each of these patients made a full neurological recovery.

Early Basic Life Support and RSI are key interventions in this group. This data should inform decisions around activating first responders and Pre Hospital Emergency Medicine teams for head injuries.

**Reference**

Atkinson JL. The neglected prehospital phase of head injury: apnea and catecholamine surge. Mayo Clin Proc. 2000 Jan;75(1):37-47.

## A12 Time well spent? Improving the performance improvement programme in a busy Trauma Unit

### N Roberts, D Ashton-Cleary, M Jadav

#### Royal Cornwall Hospital, Truro, UK

##### **Correspondence:** N Roberts (robertsneil88@gmail.com) – Royal Cornwall Hospital, Truro, UK

**Background**

Performance improvement (PI) programmes are associated with improved mortality and morbidity in Major Trauma Centres and represent a key part of the maturation process key to achieving optimum outcomes [1,2]. A weekly case discussion meeting has existed at the authors’ Trauma Unit (TU) since inception in 2011, with no formal process for tracking progress or ongoing problems. This audit aimed to elicit recurrent or common themes as targets for improvement, and to suggest improvements to make the PI programme more effective.

**Methods**

Retrospective review of slide-sets from weekly PI meetings between January 2011-January 2015. Thematic analysis of problems and learning points recorded in meeting minutes. Themes ranked by ‘number of meetings discussed’ then broken down as ‘overall’ and by year to track progress.

**Results**

174 of potential 211 slidesets available for review. 3 weeks had no cases for discussion. 59 slidesets had no learning points or problems recorded. 42 themes identified, discussed at between 1-35 meetings (mean 7.6, median 4.5). Overall most common themes: Delays to CT scan (35), delayed/lack of trauma call (26), poor/no ATMIST pre-alert (21), delays to CT report (20). Some themes were consistent problems (eg lack of trauma call). Others (eg delays to CT report) showed improvement.

**Conclusions**

Though progress has been made in some areas, many problems remain consistent despite being discussed each week. Better tracking of these may allow more effective improvement and more rapid maturation of the TU to achieve better outcomes. A self-auditing spreadsheet, based on the themes identified, with named persons taking responsibility for action points each week, has since been implemented with the aim of making the PI programme more effective. Other TUs could consider using such simple tools to enable ongoing quality improvement.

**References**

1. Liberman M, Mulder D, Jurkovich G et al. The association between trauma system and trauma center components and outcome in a mature regionalized trauma system Surgery 2005;137:647-658

2. Davenport RA, Tai N, West A et al. A major trauma centre is a specialty hospital not a hospital of specialties. Br J Surg. 2010;97:109-117.

## A14 Clinical significant and outcome of pulmonary contusions in patients with blunt chest trauma

### Ismail Mahmood, Ayman El-Menyar, Basil Younis, Ahmed Khalid, Syed Nabir, Mohamed Nadeem Ahmed, Omer Al-Yahri, Hassan Al-Thani

#### Department of Surgery, Trauma Surgery, Hamad General Hospital, Doha, Qatar

##### **Correspondence:** Ismail Mahmood (ismailyousifmah@yahoo.com) – Department of Surgery, Trauma Surgery, Hamad General Hospital, Doha, Qatar

**Background:** Currently, the use of Computed tomography (CT) is increasing in trauma patients to identify pulmonary contusions (PC). We aimed to investigate whether the size of PC has implications on the outcome in blunt trauma patients.

**Patients and methods:** Patients with PC were identified from the database registry from a Level 1 trauma center over the 3 years period ending at January 2015. The primary outcome of the study was the development of ARDS, pneumonia and mortality. PC was quantified by CT scan measurement. Patients’ demographics, injury severity, associated injuries, indication of mechanical ventilation, need for blood transfusion, base deficit, lactate level, complications and outcome.

**Result:** We identified 226 patients with PC, of them 93 % were male, mean age was 35.2 years. Associated injuries included head injury (44.2 %), abdominal injury (36.8 %), long bones fracture (35.3 %), hemothorax (38.5 %) and pneumothorax (30.1 %). Mean ISS was 20.5 ± 10.4 and chest AIS was 3.06 ± 1.5. Percent of contusion was 7.1 % (ranged 0.2-59.5). Serum lactate 3.24 ± 1.85, base deficit 4.9 ± 3.9, need of blood transfusion (33.6 %), and mechanical ventilation (42.9 %). Outcomes included chest infection (27 %), ARDS (12.4 %), and mortality (6.6 %). The majority of deaths were due to head trauma. There was a significant association between the size of PC (P = 0.003), Injury severity score (p = 0.001), blood transfusion (0.001) head injury (p = 0.001), Base deficit (p = 0.001), and duration of mechanical ventilation (p = 0.001).

**Conclusion:** In patients with blunt chest trauma, the size of PC plays an important prognostic role in association with severity of injury, blood transfusion requirement, associated head injury, the need and duration of mechanical ventilation.

**References**

1. Marc A. Blunt Pulmonary Contusion: Admission Computed Tomography Scan Predicts Mechanical Ventilation .J Trauma. 2011;71: 1543–1547.

2. Jaap Deunk, The Clinical Outcome of Occult Pulmonary Contusion on Multidetector-Row Computed Tomography. J Trauma. 2010;68: 387–394.

## A15 Plastics operative workload in major trauma centres: a national prospective survey

### Katie Young^1^, Susan A. Hendrickson^1^, Georgina Phillips^1^, Matthew D. Gardiner^2^, Shehan Hettiaratchy^1^

#### ^1^Major Trauma Centre, St Marys Hospital, Imperial College NHS Healthcare Trust, London, UK; ^2^Imperial College, London, UK

##### **Correspondence:** Katie Young (katie.c.e.young@gmail.com) – Major Trauma Centre, St Marys Hospital, Imperial College NHS Healthcare Trust, London, UK

**Background**

The introduction of major trauma centres (MTCs) in the UK in 2012 has resulted in a 63 % improvement in probability of survival of trauma [1]. The recent National Peer Review of Major Trauma [2] has found inadequate orthoplastic services and poor compliance with open lower limb fracture management guidelines across the network. The impact of the major trauma network on plastic surgical operative workload has not previously been described. This study aims to quantify this workload to help guide service design, postgraduate training, and workforce provision.

**Methods**

A prospective, multicentre study was performed. All Trauma Audit & Research Network-eligible patients presenting to eleven MTCs in England over a three-month period were identified. Demographics of all patients, and operative data for those requiring plastic surgery were obtained.

**Results**

A total of 2963 patients were admitted to the eleven MTCs within the study period. 1,582 required surgical intervention, of which 227 (14.3 %) required plastic surgery. A third of these required more than one theatre visit.

A total of 847 plastic surgical procedures were performed. Wound debridement accounted for 27.2 % and free-flaps for 4.4 %. The most frequently operated on body regions were the upper (30.9 %) and lower (50.4 %) extremities. Consultant plastic surgeons performed 61 % of the procedures.

**Conclusion**

Major trauma is a significant yet often unrecognised aspect of the plastic surgeon’s workload. This data demonstrates that the plastic surgery skill set is required in this patient population.

To ensure on-going quality care, enable workforce planning, and highlight training requirements, the important role which plastic surgery plays in the management of major trauma patients must be acknowledged.

**References**

1. TARN Press Release. https://www.tarn.ac.uk/Content.aspx?c=3477. Accessed 22.10.2015.

2. National Peer Review Report: Major Trauma Networks 2013/2014. An overview of the findings from the 2013/2014 National Peer Review of Trauma Networks in England National Peer Review Programme;2014.

## A16 A survey to assess the accuracy of estimating height by pre-hospital clinicians: can we reliably predict those most at risk of serious injury?

### Alexandra Alice Crossland, Anthony Hudson

#### St George’s Accident and Emergency Department, St George’s Hospital Medical School (University of London), Tooting, London, UK

##### **Correspondence:** Alexandra Alice Crossland (m1301443@sgul.ac.uk) – St George’s Accident and Emergency Department, St George’s Hospital Medical School (University of London), Tooting, London, UK

**Background**

Falls are a major cause of traumatic injuries only coming second to road traffic accidents [1]. This is reflected in the UK with London's Air Ambulance responding to 307 incidents regarding falls from height within a 6 month period [2].

When attending falls pre-hospital clinicians estimate the height to help assess the severity of injury mechanism. Likewise regional major trauma triage tools require pre-hospital clinicians to base triage decisions on estimated height. This identifies patients at risk of major injury and allows subsequent steps to be taken (e.g. major trauma team activation, adequate imaging and dispatch of enhanced care teams) [3].

At present no formal training of height estimation is incorporated into paramedical training. With the criteria for escalation of patient care dependent on factors such as height there should be an effective, precise way for this to be calculated so specific and correctly directed care is provided, identifying those at risk of substantial and life threatening injuries.

**Method**

A questionnaire was used, 8 different height scenarios were measured with a 30metre measuring tape and photographed. The person was given 1 minute to estimate the height for each picture.

**Results**

Results show there is no consistency in accurately estimating heights by non-qualified (paramedical students) and qualified pre-hospital clinicians. Factors such as job profession, the length of time within that profession and years of pre-hospital experience also show no correlation with the accuracy in estimation of different heights.

**Conclusion**

This study suggests better training may improve accurate triage in patients that have suffered a fall from height. Additional benefits might include better use of resources, improved triage and access to appropriate imaging.

**References**

1. World Health Organisation. Falls. [Online] Available from: http://www.who.int/mediacentre/factsheets/fs344/en/ [Accessed 29th June 2015].

2. London Air Ambulance. London’s Air Ambulance reveals trauma stats. [Online] Available from: http://londonsairambulance.co.uk/our-service/news/2014/09/trauma-statistics-revealed-1-januaryto-31-august-2014 [Accessed 29th June 2015].

3. Brown S, Cooke M, Fisher J. UK Ambulance Services Clinical Practice Guidelines.4th edition. Slovenia:Class Professional Publishing; 2013

## A17 An audit of the cause, outcome and adherence to treatment Standard Operating Procedure (SOP) for all traumatic cardiac arrests at a Helicopter Emergency Medical Service over a 12-month period

### Nicholas C Brassington^1^, Anthony Hudson^2^, Emily McWhirter^3^

#### ^1^St George’s University of London, London, UK; ^2^Emergency Department, St George's Hospital, London, UK; ^3^Kent Surrey and Sussex Air Ambulance Trust Helicopter Emergency Medical Service (KSSAAT HEMS), Redhill, UK

##### **Correspondence:** Nicholas C Brassington (m1004223@sgul.ac.uk) – St George’s University of London, London, UK

**Background:** Patients in Southeast England who suffer an out-of-hospital traumatic cardiac arrest (TCA) often have a KSS HEMS crew dispatched to support the care they receive from the local ambulance service. This group of patients carry a very poor prognosis, with a published survival rate of between 0 - 3.7 % [1-4].

**Method:** This was a retrospective review of the available data from the trauma database, HEMSBase. All patients who received treatment following a TCA between 01/05/2014–01/05/2015 were identified (**n = 79**). Each patient record was interrogated to find out the proportion of patients who achieved Return of Spontaneous Circulation (ROSC). The record of all assessments and interventions carried out were scrutinised against the TCA treatment algorithm.

**Results:** Road Traffic Collisions (RTCs) accounted almost half (47) of all TCAs. Of these, 20 involved a motorcyclist, 14 involved pedestrians and 13 involved the passenger / driver of a car. 13 patients suffered a TCA following a fall from height, 6 were due to hanging and 3 were due to stabbing. A further 32 patients were treated for a TCA due to other causes such as shooting, electrocution and crush injuries. Full compliance with the TCA SOP was achieved in 85 % of patients with a further 10 % deemed to be a justifiable deviation by a senior HEMS consultant.

**Discussion/Conclusion:** RTCs remain the largest overall cause of TCA (49 %) and those suffering isolated head and/or neck injuries carried the best outcome (33 %) of patients achieving ROSC. These findings could be useful in prompting accident prevention, as motorcycle riders are responsible for a small percentage of road miles but a high proportion of mortality on the road or prompting early dispatch of a HEMS crew. Adherence to the treatment SOP was high (95 %) but this should be monitored to maintain the high standards of compliance.

**References**

1. Battistella FD, Nugent W, Owings JT, et al. Field triage of the pulseless trauma patient. Arch Surg. 1999; 134:742-745

2. Stockinger ZT, McSwan NE. Additional evidence in support of withholding terminating cardiopulmonary resuscitation for trauma patients in the field. J Am Coll Surg. 2004; 198:227-231.

3. Shimazu S, Shatney CH. Outcomes of trauma patients with no vital signs on hospital admission. J Trauma. 1983 Mar;23(3):213-6.

## A18 Should we “stay-and-play? A study of patient physiology in Norwegian Helicopter Emergency Services

### Bjørn O Reid^1^, Marius Rehn^2,3,4^, Oddvar Uleberg^1^, Andreas J Krüger^1,2^

#### ^1^Department of Emergency Medicine and Prehospital Services, St. Olavs Hospital, Trondheim, Norway; ^2^Department of Research and Development, Norwegian Air Ambulance Foundation, Drøbak, Norway; ^3^London's Air Ambulance, Barts Health Trust, Whitechapel, London, UK; ^4^Field of Pre-hospital Critical Care, Network of Medical Sciences, University of Stavanger, Stavanger, Norway

##### **Correspondence:** Bjørn O Reid (bjorn.ole.reid@stolav.no) – Department of Emergency Medicine and Prehospital Services, St. Olavs Hospital, Trondheim, Norway

**Background**

When to” stay-and-play”, “load-and-go” or “run-and-play” may prove to be a challenge for the pre-hospital practitioner [1]. Improvement in patient physiology could be interpreted as a surrogate for the effect of pre-hospital management. Utilizing the Mainz Emergency Evaluation Score (MEES), we aim to analyze the physiological profile of patients attended by physician-staffed Norwegian Helicopter Emergency Services (HEMS) and whether there was a relationship with on-scene-time expenditure and advanced procedures.

**Method**

Six Norwegian HEMS bases prospectively registered patient data during two summer and two winter weeks in 2009-2010. MEES scores include the following variables: Glasgow Coma Scale, heart rate, respiratory rate, systolic blood pressure, arterial oxygen saturation, heart rhythm and pain. The score was used to classify the degree of physiological derangement and physiological changes during patient contact.

**Results**

290 patients-on-scene were included in the study, of which 61 % were male. Using the study criteria, 43 % were considered severely ill or injured, and 29 % had severely deranged physiology. Of the latter 67 % were medical patients and 31 % were trauma. 153 advanced procedures were performed, the most common being advanced airway management (16 %) and defibrillation (8.6 %). By comparing first-contact and end-of-care MEES scores, we found that 33 % of patients improved, 66 % were unchanged and 1 % deteriorated. In patients receiving advanced medication and procedures, 51 % and 42 % improved respectively with none deteriorating. With increasing on-scene-time up to 40 minutes, we showed that a greater proportion of patients improved and fewer deteriorated.

**Conclusion**

We have shown that it is possible to restore deranged physiology in the pre-hospital setting, also when this implies prolonging on-scene times or commencing advanced treatment. This may be prudent, especially with increased transport times to treatment facilities.

**Reference**

1. Smith RM, Conn AK: Prehospital care - scoop and run or stay and play? *Injury* 2009, 40 Suppl 4:S23-26.

## A19 Training in resuscitative thoracotomy: have we cracked it? A survey of higher Emergency Medicine trainees in London

### Cara Jennings^1^, Yasmin Kapadia^2^, Duncan Bew^3^

#### ^1^Emergency Department, St. Thomas’ Hospital, London, UK; ^2^Emergency Department, King's College Hospital, London, UK; ^3^Trauma and Emergency Surgery, King’s College Hospital, London, UK

##### **Correspondence:** Cara Jennings (carajennings@doctors.org.uk) – Emergency Department, St. Thomas’ Hospital, London, UK

**Introduction:** Resuscitative thoracotomy (RT) is within the capabilities of Emergency Medicine (EM) physicians. Survival rates for RT in the Emergency Department (ED) was quoted as 21 % in a recent study [1]. Since publication of ‘Emergency Thoracotomy: “How to do it”’ in 2005 [2] a range of practical courses have been developed. Research suggests practical training to be superior to theoretical training [3]. The Royal College of Emergency Medicine (RCEM) curriculum 2015 states higher trainees should have knowledge and skills in RT.

**Aim:** To compare clinical and training experience of London higher trainees in EM in 2015 with that of a similar cohort in 2005.

**Methods:** A questionnaire exploring the experience of RT was distributed to EM higher trainees in 2005 and 2015.

**Results:** In 2015 there were 62 respondents out of 106 trainees. 37 % were working in a Major Trauma Centre, 29 % had a cardiothoracic registrar on site. 95 % had theoretical training and 44 % had practical training. 84 % knew the indications for RT. 73 % would use a clamshell approach. 56 % had witnessed an RT. 75 % were in ED. 84 % in a MTC. 22 % were successful. 45 % of respondents felt able to perform RT. In 2005 81 out of 121 EM SpRs completed the survey, 64 % felt able to perform an RT. 46 % had received training and 42 % had exposure to the procedure.

**Conclusion:** The study suggests more trainees are being trained in RT. The majority had received theoretical training in 2015 and knew the indications. Practical training in RT had the strongest correlation with confidence in performing the procedure. Less than half of higher trainees have had practical training in 2015. Inclusion of RT in the RCEM 2015 curriculum will hopefully lead to the development and expansion of affordable and accessible training aimed at the ED physician.

**References**

1. Seamon MJ et al. An evidence-based approach for patient selection for ED thoracotomy: A practice management guideline from the Eastern Association for the Surgery of Trauma. Trauma Acute Care Surg. 2015 Jul; 79(1):22-24

2. Wise D et al. Emergency Thoracotomy: “how to do it.” Emerg Med J; 2005 Jan;22(1):22-24

3. Chapman DM et al. Open thoracotomy procedural competency: validity study of teaching and assessment modalities. Ann Emerg Med. 1996 Dec: 28(6): 641-7

## A20 London’s Air Ambulance (LAA): 25-years of drownings in an urban environment

### Jenny Townsend, Tom P Hurst, Elizabeth A Foster

#### Helicopter Emergency Medical Service, Barts Health NHS Trust, London, UK

##### **Correspondence:** Jenny Townsend (jtownsend@doctors.org.uk) – Helicopter Emergency Medical Service, Barts Health NHS Trust, London, UK

**Background**

Globally, drowning is the second leading cause of unnatural death after road traffic injuries. (1) The Royal Society for the Prevention of Accidents state there were 381 accidental drownings across the UK in 2013. (2) The aim of this study is to describe the urban drownings attended by LAA over the last 25 years.

**Method**

A retrospective review of the pre-hospital patient database was performed from 1990-2015 to identify all patients who were assumed to be victims of drowning / submersion / immersion. Patient demographics, water type, presenting complaint (cardiac arrest / respiratory distress etc.), pre-hospital interventions and patient destination were captured.

**Results**

Of the 32,995 patients treated by LAA within the study period, 271 (1 %) patients met the study criteria for inclusion. A total of 13 records were excluded from the final analysis due to incompletion of key data. 258 patient records were analysed. Almost three-quarters of patients were male (72 %, n = 185), with a mean age of 33 and median age of 32 (Range = 1-86 years). Children (age < 16 years) accounted for 38 % of the overall study population. In the urban environment people drowned in: Rivers 81(32 %); Swimming pools 78(30 %); Baths 28(11 %); Canals 27(10 %); Lakes & Ponds 24(9 %); Other 20(8 %).

29 % (n = 76) had CPR and 6 % (n = 15) had rescue-breaths only during the pre-hospital phase. 39 % (n = 115) required pre-hospital tracheal intubation. 17 % (n = 44) died at scene.

**Conclusion**

We report the largest UK series of drownings in an urban environment. Children account for over one third of these drownings. Drowned patients requiring LAA attendance are frequently in need of advanced interventions.

References

1) Szpilman D, Bierens J.J.L.M et al. Current Concepts Drowning: N Eng J Med 2012, 366; 2102-2110.

2) The Royal Society for the Prevention of Accidents Drowning Statistics http://www.rospa.com/leisure-safety/statistics/drowning/

## A21 Live patients in trauma simulation – more than just simulation on a shoestring?

### Thomas B Brown, Joseph Collinson, Christopher Pritchett, Toby Slade

#### Emergency Department, Royal Cornwall Hospital, Truro, UK

##### **Correspondence:** Thomas B Brown (thomas.brown@doctors.org.uk) – Emergency Department, Royal Cornwall Hospital, Truro, UK

**Background**

Simulation training is increasingly used within the Emergency Medicine curriculum with various peer-reviewed journals promoting simulation as a useful educational tool^1,2^. High-fidelity mannequins can demonstrate certain clinical signs but considerable barriers remain, particularly a lack of human interaction. We suggest that a live patient during a trauma simulation teaching series could both create more realistic scenarios, and develop non-technical skills between the multi-disciplinary trauma team.

**Method**

A live clinically trained actor, rather than a high-fidelity mannequin, acted as the trauma patient in three trauma simulations within the Royal Cornwall Hospital Emergency Department. Each scenario lasted approximately 20 minutes, and was followed with a de-briefing session. Feedback was collected from 12 Emergency Medicine doctors using visual analogue scale questionnaires.

**Results**

100 % found interacting with a live patient a positive learning experience. 92 % stated it enhanced realism and the learning experience versus a high-fidelity mannequin. 100 % felt a live patient was superior for non-technical skills. 75 % felt a mannequin was better for developing procedural skills. 16.7 % felt more pressured when managing a live patient compared to a high-fidelity mannequin.

**Discussion**

Our data suggests that interaction with a live, simulated patient creates a realistic trauma simulation providing the opportunity to develop non-technical skills within the multi-disciplinary team. We appreciate that particular scenarios may not be suitable for this approach. However, many scenarios can be effectively utilized, such as kit-off and log-roll, essential in trauma cases. A potential drawback of this approach is the difficulty in performing invasive procedures. Interestingly, we rarely perform such procedures on high-fidelity mannequins in any case, further strengthening the case for live patient simulation.

**References**

1) Ten Eyck RP. Simulation in emergency medicine training. Pediatr Emerg Care 2011, 27(4); 333-341

2) Gjerraa K, Møller TP, Østergaard D. Efficacy of simulation-based trauma team training of non-technical skills. A systematic review. Acta Anaesthesiol Scand 2014, 58(7); 775-787

## A22 Collecting core data in pre-hospital critical care using a consensus based template

### Kristin Tønsager^1,2^, Marius Rehn^1,3^, Kjetil G.Ringdal^1,4^, Andreas J.Krüger^1,5^

#### ^1^The Norwegian Air Ambulance Foundation, Drøbak, Norway; ^2^Department of Anaesthesiology and Intensive Care, Stavanger University Hospital, Stavanger, Norway; ^3^London’s Air Ambulance, Barts Health, London, UK; ^4^Department of Anaesthesiology, Vestfold Hospital Trust, Tønsberg, Norway; ^5^Department of Anaesthesia and Acute Care, St. Olavs Hospital, Trondheim, Norway

##### **Correspondence:** Kristin Tønsager (kristin.tonsager@norskluftambulanse.no) – Department of Anaesthesiology and Intensive Care, Stavanger University Hospital, Stavanger, Norway

**Background**

Uniform documentation is fundamental for medical research. Lack of documentation is a well-known limitation for research regarding emergency medicine, especially for retrospective registry studies [1]. Physiological parameters are reported to be the most often missing variables [1, 2]. In 2011 a consensus-based template for documenting and reporting in physician-staffed pre-hospital critical care services was designed, aiming to provide a reporting system that allowed identification of key factors related to favourable outcomes across different international physician-staffed Emergency Medical Services (p-EMS) [3]. The present study evaluated the feasibility of collecting patient and system level data by using the template.

**Methods**

Template data was collected prospectively for six weeks (20.01. – 09.03.14) by 16 physicians from four of the 12 Norwegian p-EMS bases; Trondheim, Ålesund, Bergen and Stavanger. Physicians who were motivated and expected to achieve a high data collection compliance were recruited. The importance of maximal effort to collect all variables during the study period was emphasized.

**Results**

Data from 177 missions were submitted. Of these 49 (28 %) missions were excluded because of no patient encounter, leaving 128 (72 %) missions eligible for further analyses. Results showed that 90 % of all template data were collected. 85 % of all physiological parameters (respiratory rate, cardiac rhythm, heart rate, pulse oximetry and systolic blood pressure) were collected. Completeness rates did not change for seriously injured patients (p = 0,08) but decreased for short missions where patients were cared for less than 20 minutes (p < 0,05).

**Conclusion**

This study of the ability of the p-EMS to collect data by using a template demonstrates that completeness rates can be high when efforts are optimized, even for physiological parameters. The template is feasible for data collection.

References

1. Laudermilch DJ, Schiff MA, Nathens AB, Rosengart MR: **Lack of emergency medical services documentation is associated with poor patient outcomes: a validation of audit filters for prehospital trauma care.***J Am Coll Surg* 2010, **210:**220-227.

2. di Martino P, Leoli F, Cinotti F, Virga A, Gatta L, Kleefield S, Melandri R: **Improving vital sign documentation at triage: an emergency department quality improvement project.***J Patient Saf* 2011, **7:**26-29.

3. Kruger AJ, Lockey D, Kurola J, Di Bartolomeo S, Castren M, Mikkelsen S, Lossius HM: **A consensus-based template for documenting and reporting in physician-staffed pre-hospital services.***Scand J Trauma Resusc Emerg Med* 2011, **19:**71.

## A23 Prehospital interventions before and after implementation of a physician staffed helicopter

### Rasmus Hesselfeldt, Sandra Wulffeld, Asger Sonne, Lars S. Rasmussen, Jacob Steinmetz

#### Department of Anaesthesia, Centre of Head and Orthopaedics, Rigshospitalet, University of Copenhagen, Copenhagen, Denmark

##### **Correspondence:** Rasmus Hesselfeldt (hesselfeldt@hotmail.com) – Department of Anaesthesia, Centre of Head and Orthopaedics, Rigshospitalet, University of Copenhagen, Copenhagen, Denmark

Background

The implementation of a physician staffed Helicopter Emergency Medical Services (HEMS) in eastern Denmark was associated with an increase in the proportion of severely injured trauma patients transported directly to a level 1 trauma centre and mortality was reduced significantly[1].

This study aims to compare the number of advanced interventions provided on-scene or en-route to the hospital before and after the implementation.

Method

This was a post-hoc analysis of a prospective observational study[1]. We included trauma patients in the period December 1^st^, 2009 to April 30, 2010 (before HEMS implementation) and from May 1^st^, 2010 to April 30, 2011 (after the HEMS implementation) in a pre-specified geographical area.

In patients with an Injury Severity Score (ISS) > 3, transported by any Emergency Medical Service, we compared the proportion of patients having at least one of 13 predefined advanced interventions.

Results

A total of 735 patients (after n = 546, before n = 189) were included. No significant differences in demographic data, injury severity score, or case mix were observed. A significant higher proportion of patients had one or more advanced prehospital interventions done after (33.7 %, n = 184) vs. before (21.2 %, n = 40) HEMS implementation (p < 0.01).

In patients with a Glasgow Coma Score <9 and/or a ‘Head’ Abbreviated Injury Score > 3, tracheal intubation was done prior to arrival at the hospital in 48.6 % (35/72) after the HEMS was part of the system vs. 28.1 %, (9/32) p = 0.05. Needle decompression, chest tube, or finger thoracotomy were uncommon in both groups (7 vs. 0). Analgesics were given in 21.8 % after HEMS implementation vs. 11.1 %, p < 0.01.

Conclusion

More patients received advanced prehospital interventions after implementation of a physician staffed HEMS.

Reference

1. Hesselfeldt R, Steinmetz J, et al. Impact of a physician-staffed helicopter on a regional trauma system: a prospective, controlled, observational study**.** Acta Anaesthesiol Scand 2013, 57:660–668.

## A24 Duration of ventilation following prehospital drug assisted intubation; a retrospective review

### Thomas J Renninson^1^, Nadine Thomson^2^, Harvey Pynn^2^, Timothy J Hooper^2,3^

#### ^1^Department of Anaesthetics, Gloucester Royal Hospital, Gloucester, UK; ^2^Great Western Air Ambulance, Filton Air Support Unit, Bristol, UK; ^3^Intensive Care Department, Southmead Hospital, Bristol, UK

##### **Correspondence:** Thomas J Renninson (tom.renninson@doctors.net.uk) – Department of Anaesthetics, Gloucester Royal Hospital, Gloucester, UK

Background

Drug Assisted Intubation (DAI) using endotracheal tubes (ETT) is part of routine practice in many physician lead prehospital care services. The use of subglottic secretion drainage (SSD) ETT in intensive care units (ICU) has been shown to decrease the incidence of ventilator-associated pneumonia^1^. This review looks at the duration of ventilation following prehospital DAI to determine whether there is a case for SSD ETT use in this setting. Additionally, it analyses the appropriateness of prehospital DAI in those who were ventilated for a short duration prior to extubation.

Methods

100 consecutive trauma patients receiving prehospital DAI were identified using a prehospital clinical database and linked to hospital records using demographics, hospital arrival time and injury pattern. The patient’s clinical course and duration of ventilation was determined from their records. Patient’s extubated within 24 hours of prehospital DAI were further analysed. Those with normal CT imaging and no further clinical intervention prior to extubation were independently reviewed by a panel of 5 consultants who were asked to decide whether DAI was indicated.

Results

Of 100 cases, 96 were linked to patient records. Of these, 43 (44.8 %) were ventilated for >72 hours via prehospital ETT and 51 (53.1 %) were extubated within 24 hours. 13 patients extubated following normal CT imaging with no further intervention were submitted for review. The panel agreed with the indication for DAI in all cases (unanimously in 9 and by majority in 4).

Discussion

42.7 % of patients ventilated for >72 hours following prehospital DAI may have benefited from the use of a SSD ETT^1^. Prehospital care units should liaise with their local ICU to determine potential clinical and cost benefits with the aim of producing a unified protocol. All patients in the study had an appropriate indication for prehospital DAI regardless of duration of ventilation.

Reference

1. Muscedere J, Rewa O, McKechnie K, Jiang X, Laporta D, Heyland DK. Subglottic secretion drainage for the prevention of ventilator-associated pneumonia: a systematic review and meta-analysis. Crit Care Med 2011, 39(8);1985-91

## A25 Non-haemorrhagic shock in trauma: a novel guideline for management in ED

### Anthony Hudson^1,2^, Jacinta Dawson^1^, Ashley Matthies^1^

#### ^1^Emergency Department, St George’s Hospital, London, London, UK; ^2^Kent, Surrey and Sussex Air Ambulance Trust, Marden, Kent, UK

##### **Correspondence:** Jacinta Dawson (jacinta.dawson@stgeorges.nhs.uk) – Emergency Department, St George’s Hospital, London, London, UK

**Background**

Trauma is a major contributor to mortality and morbidity worldwide, and the identification and management of the unstable or shocked trauma patient plays a large role in determining patient outcomes. Patients in shock have high mortality rates which correlate to the severity and duration of hypoperfusion. A systematic approach must be used to diagnose and manage causes of shock. Traditional teaching places emphasis on haemorrhage as a cause of trauma deaths, however hypotension in trauma may be multifactorial in nature or purely a result of non-haemorrhagic causes. Other causes of shock to consider and rule out before proceeding with massive transfusion include neurogenic, obstructive, cardiogenic, sepsis, toxins, and exposure. Proceeding to massive transfusion carries potential complications which have serious implications for patient outcomes particularly if transfusion is inappropriate. The underlying cause of trauma as well as the effect of injuries sustained needs to be considered.

**Method**

We have developed and are trialling a novel guideline “Management of Non-Haemorrhagic Shock in Trauma” in the Emergency Department at St George’s Hospital, London, which is a Major Trauma Centre.

**Results**

Anecdotally, this guideline has benefits in cases including: collapse from acute MI, resultant fall down stairs causing multiple injuries, element of cardiogenic shock would have responded poorly to massive transfusion; intoxicated patient with head injury, hypotension related to intoxication and hypothermia; post-arrest trauma patient with head and neck burns, rescued from fire in car – complicated by possible cyanide toxicity and carbon monoxide poisoning.

**Discussion**

We propose that this guideline is used to aid in earlier recognition of factors contributing to shock. This does not need to delay starting transfusion, but rather should help to halt unnecessary transfusion before it progresses. We aim to produce a case series to support the more widespread use of this guideline.

## A26 Patient-tailored triage decisions by anaesthetist-staffed pre-hospital critical care teams

### Morten Langfeldt Friberg, Leif Rognås

#### Pre-hospital Critical Care Services, The Central Denmark Region, Aarhus, Denmark

##### **Correspondence:** Morten Langfeldt Friberg (morten.friberg@gmail.com) – Pre-hospital Critical Care Services, The Central Denmark Region, Aarhus, Denmark

Background

In the Central Denmark Region, the emergency medical service consists of emergency medical technician (EMT)-staffed ambulances and anaesthetist- EMT-staffed pre-hospital critical care teams (1).

There are pre-defined fast-track protocols routing patients with ST-elevation myocardium infarction (STEMI) (2, 3) and stroke as well as for women in labour and children under the age of 15 to other departments than the local emergency departments (ED).

The pre-hospital critical care anaesthetists may refer critically ill and injured patients directly to specialised departments to minimise time to definitive care. The extent of this triage is not known.

Aim: to estimate the incidence of patients not covered by one of the pre-defined regional fast-track protocols but triaged by the pre-hospital critical care anaesthetists to by-pass the local adult or paediatric ED.

Method

Retrospective descriptive study.

Inclusion criteria: all patients treated by the pre-hospital critical care teams during 2013 and -14.

Exclusion criteria: patients with STEMI, stroke or in active labour.

Endpoints: incidence of pre-hospital critical care anaesthetist-provided triage, tentative diagnoses and transport destination.

Results

The pre-hospital critical care teams treated 39396 patients and diverted 989 (2.5 %) non-STEMI, non-stroke patients not in active labour directly to a specialised department.

The diagnoses “*resuscitated after cardiac arrest*” (n = 143), *“treatment and observations following road traffic accident”* (n = 105) and “*observation and treatment for an unspecified disease/condition*” (n = 78) were the most common; together they accounted for 33.0 % of all diverted patients.

943 (95.3 %) of the pre-hospital critical care team-diverted patients were diverted to a department at Aarhus University hospital.

Conclusion

Our results showed that in 1 in 40 contacts, i.e. approximately 500 times a year, the pre-hospital critical care teams in the Central Denmark Region divert critically ill and injured patients directly to a specialised department, potentially reducing time to definitive care for these patients.

References

1. Rognås L, Hansen TM, Kirkegaard H, Tønnesen E. Pre-hospital advanced airway management by experienced anaesthesiologists: a prospective descriptive study. Scandinavian journal of trauma, resuscitation and emergency medicine. 2013;21:58.

2. Sørensen JT, Terkelsen CJ, Nørgaard BL, et al. Urban and rural implementation of pre-hospital diagnosis and direct referral for primary percutaneous coronary intervention in patients with acute ST-elevation myocardial infarction. European Heart Journal. 2011;32(4):430-6.

3. Terkelsen CJ, Sørensen JT, Maeng M, et al. System delay and mortality among patients with STEMI treated with primary percutaneous coronary intervention. JAMA : the journal of the American Medical Association. 2010;304(7):763.

## A27 Anatomical accuracy and appropriate sizing of pre-hospital thoracostomies

### Jessica FG Wills^1^, Anthony Hudson^2^

#### ^1^St George’s Hospital Medical School, University of London, London, UK; ^2^Emergency Department, St George’s Hospital, London, UK

##### **Correspondence:** Jessica FG Wills (jessicawills@doctors.org.uk) – St George’s Hospital Medical School, University of London, London, UK

Background

Thoracic trauma contributes to up to 50 % of traumatic deaths^1^. Pre-hospital thoracostomy increases successful outcomes but guidelines must be followed accurately to reduce complications.

Aim

Assess compliance of KSS HEMs clinicians with the standard operating procedure (SOP) for thoracostomy using models of differing BMIs.

Method

A ‘gold standard’ anatomical location and size was determined on two male models (BMIs 21.5 and 29.4) according to the SOP. Staff performed a blind thoracostomy skills test. A retrospective search was undertaken of the KSS HEMs patient database to garner demographical information.

Results

243 thoracostomies were performed on 125 patients, 98 male and 27 female, over a 12 month period. The most common mechanism of trauma was road traffic collision, accounting for 74.4 % of presentations. In total, 51.2 % of patients were pronounced life extinct on scene.

The skills test was undertaken by 8 paramedics and 7 doctors, the majority indicating an incision site in line with the SOP; 86.7 % for BMI 21.5, 80 % for BMI 29.4. A wider range of potential spaces were proposed for the higher BMI, with estimations spread over 5 intercostal spaces compared with 3 in the lower BMI. As with location, the size of thoracostomies was largely consistent with the SOP.

Discussion

All staff knew the correct site and size for thoracostomy based on the SOP. In practice, however, there was a range of variation plus a noticeable difference between the models. A greater degree of inaccuracy was seen with the higher BMI. This may have implications for clinical practice in a population of increasing BMI. The majority of estimations for both models were clustered around the gold standard zone with abnormalities being outliers rather than the norm.

Reference

Perkins Z, Gunning M. Life-saving or life-threatening? Prehospital thoracostomy for thoracic trauma. Emerg Med J. 2007, 24;305-306.

## A28 Pre-hospital management of mass casualty civilian shootings

### Conor DA Turner^1,2^, Marius Rehn^3,4,5^

#### ^1^Barts and The London School of Medicine and Dentistry, Garrod Building, Turner Street, Whitechapel, London, E1 2AD, UK; ^2^The Institute of Pre-Hospital Care, 7-8 Philpot Lane, City of London, London EC3M 8A, UK; ^3^London’s Air Ambulance, Royal London Hospital, Whitechapel Road, London E1 1BB, UK; ^4^The Norwegian Air Ambulance Foundation, Holterveien 24, N-1441, Drøbak, Norway; ^5^Field of Pre-hospital Critical Care, University of Stavanger, Kjell Arholmsgate 41, N-4036, Stavanger, Norway

##### **Correspondence:** Conor DA Turner – The Institute of Pre-Hospital Care, 7-8 Philpot Lane, City of London, London EC3M 8A, UK

**Background** On the background of a rising threat of transnational terrorism worldwide[1], emergency response strategies are of critical importance. There is a need for information supported by evidence and experience for medical providers responding to an active shooter type major incident. This comprehensive review aims to collect and consider the indexed and non-indexed literature on the pre-hospital management of modern civilian mass shootings to guide future practice.

**Methods** Systematic literature searches of Pubmed and Scopus were conducted in conjunction with simple searches of non-indexed databases; ‘Web of Science’, ‘Open Doar’ and ‘Evidence Search’. The searches were last carried out on the 15^th^ of April 2015 and only identified those papers published after the 1^st^ of January 1980.

**Results** 472 documents were identified of which 347 were excluded based on abstract and title, 83 were excluded after full text reading leaving 42 documents with an additional 5 references for inclusion in analysis. The search yielded nineteen case reports of fifteen mass shooting events, the majority from the USA with additions from Norway, the UK and Kenya. 28 other documents found in the search were used for extraction of relevant information. Quality appraisal demonstrated a considerable variability in reporting, furthermore there is limited data on mass shootings globally. The acronym, THREAT which stands for, Threat suppression, Haemorrhage control, Rapid Extraction from scene, Assessment by medical providers and Transport to definitive care, incorporates the major elements of a successful pre-hospital response to these incidents [2].

**Conclusions** Mass casualty civilian shootings represent an infrequent but recurring challenge to emergency services with distinct management challenges. Some key themes were identified to improve future practice. The value of tactical emergency medical support[3], improved cross-service education on effective haemorrhage control[2], the benefits of senior triage operators and the need for regular mass casualty incident simulation.

**References**

1. Sandler T, Arce DG, Enders W: *The Challenge of Terrorism*. 2008:1–95.

2. Jacobs LM, Wade DS, McSwain NE, Butler FK, Fabbri WP, Eastman AL, Rotondo M, Sinclair J, Burns KJ: The Hartford Consensus: THREAT, A Medical Disaster Preparedness Concept. *J Am Coll Surg* 2013, 217:947–953.

3. Autrey AW, Hick JL, Bramer K, Berndt J, Bundt J: 3 Echo: Concept of Operations for Early Care and Evacuation of Victims of Mass Violence. *Prehosp Disaster Med* 2014, 29(August):421–428.

## A30 The prevalence of alcohol-related trauma recidivism: a systematic review

### James Nunn^1^, Mete Erdogan^2^, Robert S. Green^2,3^

#### ^1^Dalhousie University Medical School, Halifax, Nova Scotia, Canada; ^2^Trauma Nova Scotia, Halifax, Nova Scotia, Canada; ^3^Department of Critical Care Medicine, Dalhousie University, Halifax, Nova Scotia, Canada

##### **Correspondence:** Robert S. Green (greenrs@dal.ca) – Department of Critical Care Medicine, Dalhousie University, Halifax, Nova Scotia, Canada

Background:

Recurrent hospital admission for separate incidents of traumatic injury is known as trauma recidivism. Although alcohol use is a known risk factor for injury and has been associated with trauma recidivism, the scale of alcohol-related trauma recidivism has not been well described. The objective of this review was to determine the proportion of trauma recidivism related to alcohol use. Our secondary objective was to evaluate the association between alcohol and trauma recidivism.

Method:

A search of four databases (MEDLINE, Embase, CINAHL, Web of Science) from inception until June 2015 yielded 1839 records for screening. Only primary studies that reported repeated admissions to a hospital or trauma center for traumatic injuries specifically related to alcohol use were included. Descriptive statistics were used to assess study characteristics and the prevalence of trauma recidivism related to alcohol use. An aggregate weighted estimate of alcohol-related trauma recidivism was calculated.

Results:

A total of 11 studies met all inclusion criteria. Overall, there were 3350 trauma recidivists among included studies. The proportion of trauma recidivists with evidence of alcohol use on admission ranged from 26.7 % to 76.9 % (median 47.6 %). The aggregated sample produced a weighted estimate of 41.0 % (1372/3350) for alcohol-related trauma recidivism. In four studies, the association between alcohol and trauma recidivism was examined; all four found a positive association between alcohol use and repeated admission for traumatic injury. Studies varied considerably in design, trauma populations, recidivism periods, and definitions for positive alcohol on admission.

Conclusion:

Evidence suggests that 41.0 % of trauma recidivism is related to use of alcohol. Due to methodological limitations among included studies, this may underestimate the actual prevalence of alcohol-related trauma recidivism.

## A31 Development of a hospital-wide program for simulation-based training in trauma care and management

### Samuel Minor^1,2^, Mete Erdogan^3^, Kathy Hartlen^3^, Robert S. Green^1,3^

#### ^1^Department of Critical Care Medicine, Dalhousie University, Halifax, Nova Scotia, Canada; ^2^Division of General Surgery, Queen Elizabeth II Health Sciences Centre, Halifax, Nova Scotia, Canada; ^3^Trauma Nova Scotia, Halifax, Nova Scotia, Canada

##### **Correspondence:** Robert S. Green (greenrs@dal.ca) – Trauma Nova Scotia, Halifax, Nova Scotia, Canada

Background:

The Queen Elizabeth II Health Sciences Centre (QEII HSC) in Halifax is a Level I trauma center that provides tertiary care services to the province of Nova Scotia (population 940,592) and quaternary care services to Atlantic Canada (population > 2.4 million). There are three dedicated simulation facilities at the hospital: the Emergency Medicine Simulation Bay is designed to mirror a trauma room and provides training using high fidelity simulators and clinical grade cadavers; the Atlantic Health Training and Simulation Centre includes mannequin-based simulators, a debriefing room, and a control room; and the Skills Center for Health Sciences provides a place for trainees and professionals to learn and practice surgical techniques.

Method: This was a descriptive study of the development of an inter-professional hospital-wide environment for training medical students, residents, physicians and hospital staff using trauma-based simulation.

Results:

Training activities between 2013 and 2015 included “in situ” simulations of trauma activations using a sim man; these mock exercises involved assessing the “patient” in the trauma bay and then taking them to the operating room. In 2014, a Trauma Boot Camp for residents was held and involved a surgical skills lab for the development of advanced trauma-related surgical interventions.

Discussion:

Simulation-based trauma training offers a “controlled” environment for individuals from multiple specialties to safely acquire and practice an array of skills and learn how to work together as a team during critical situations. The integration of trauma simulation into medical student curriculum, residency training, and continuing medical education has great potential to enhance patient safety, reduce medical errors, and allow for the systematic evaluation of various competencies. Further research is required to validate long-term retention of skills and knowledge, to evaluate the effectiveness of simulation-based training on performance, and to assess the impact of simulation training on trauma patient outcomes.

## A32 Out of Hospital Cardiac Arrests (OOHCA); lessons from Hollywood

### Ruth Bird^1^, Rachael L. Grupping^2^

#### ^1^ST3 Anaesthetist Barts Heart Centre, London, UK; ^2^FY2 Royal Victoria Hospital, Dundee, Scotland, UK

##### **Correspondence:** Rachael L. Grupping (rachael.grupping@nhs.net) – FY2 Royal Victoria Hospital, Dundee, Scotland, UK

In the UK there are 30,000 OOHCA/year, survival to discharge is 8.6 %. The frequency of post OOHCA mortality and morbidity in Hollywood films seemed disproportionate. Our aim was to determine if these outcomes were likely and if anything could be learnt from Hollywood.

Method

Statistics taken from the Rhesus Council UK (RCUK) 2013-2014. Inclusion criteria for our retrospective study group; OOHCA with resuscitation attempted at scene. Data was analysed looking at the mechanism of arrest, underlying rhythm, whether CPR was in progress from the time of arrest and whether drugs and electricity were used. 7

Statistical analysis was performed using SPSS.

Results

RCUK statistics show 20 % have a shockable rhythm, bystander CPR in progress in 43 % of OOHCA, defibrillation in 1.74 %. In Hollywood movies, bystander CPR rates were 92.30 %, 30 % had a shockable rhythm, all successfully defibrillated. Hollywood’s overall survival rate was 92.30 %, 70 % had a traumatic mechanism compared to 4.7 % in RCUK data. 88 % of traumatic arrest patients survived, as opposed to 5.6 % (RCUK) and 100 % achieved good neurological outcome, compared to 1.6 % (RCUK). Medical causes accounted for 30 %.

Fishers exact test showed a statistical significance for survival between the two groups (p < 0.05).

Bystander CPR, early defibrillation and urgent transfer to hospital for conclusive management and robust post return of spontaneous circulation care greatly improve survival. No film stars required advanced airway intervention or prolonged hospital stay, highlighting the discrepancy between reality and film.

Cardiac arrests in films occurred mostly in young physiologically fit individuals, combined with early CPR, good response times, and early defibrillation may partially explain this statistical difference. Hollywood’s hugely successful outcome statistics for traumatic arrest are inexplicable, probably due to artistic licence. Hollywood are commended for early CPR, however, focus should be on improving quality of chest compressions and adherence to advanced life support protocols.

References

1. Rhesus Council UK (RCUK) 2013-2014 URL: https://www.resus.org.uk/quality-standards/

2. National Cardiac Arrest Audit 2013-2014, URL: https://www.icnarc.org/Our-Audit/Audits/Ncaa/Reports/Access-Our-Data/2015/10/15/Key-Ncaa-Statistics-201415

## A33 Mechanism of injury as a predictor of severity of injury in road traffic collisions: a literature review

### Amelia M. Stacey^1,2^, Marius Rehn^3^, David J. Lockey^3^

#### ^1^Barts and the London School of Medicine and Dentistry, London, UK; ^2^Institute of Prehospital Care, London, UK; ^3^London’s Air Ambulance, London, UK

##### **Correspondence:** Amelia M. Stacey (amelia.stacey@hotmail.co.uk) – Institute of Prehospital Care, London, UK

**Background**: Mechanism of injury has long been used to predict injury patterns by medical teams. As prehospital care has progressed, trauma triage tools have been developed to improve appropriate triaging to specialist trauma centres that deal with severe injuries. Vehicle crash safety design has advanced a great deal in recent times and this literature review aimed to establish which mechanisms of injury are currently used to triage trauma, whether the selected mechanisms of injury are reliable indicators of severe injury and if they should continue to be included in major trauma triage protocols.

**Method:** Trauma triage guidelines were gathered from English, American and Australian trauma network websites, eighteen in total. The three most prevalent vehicular mechanisms of injury that appeared in these protocols were identified and medical databases were searched for relevant papers and conclusions drawn.

**Results:** Vehicle rollover, ejection from the vehicle and death in the same passenger compartment were the three most common vehicular mechanisms of injury mentioned. 13 papers were found relevant for ejection from a vehicle, 31 papers for vehicle rollover and 30 for death in the same passenger compartment. Ejection carried a high risk of severe injury. A vehicle rollover, despite seeing the highest rate of cervical spine damage, was found to be a poor indicator of severe injury, especially in belted individuals. Death of an occupant in the same passenger compartment was multifactorial in causation and the best predictor of severe injury of those studied.

**Conclusions:** The role of mechanism of injury in ascertaining injury severity faces an uncertain future, with evidence both for and against looking at the mechanism found. Currently, ejection and death of an occupant in the same passenger compartment should remain in trauma triage protocols, but the rollover mechanism is not strongly correlated enough to severe injury to be included.

**References**

1. Boyle MJ, Smith EC, Archer F. Is mechanism of injury alone a useful predictor of major trauma? Injury 2008 Sep, 39(9):986–92.

2. Lerner E, Shah M, Cushman J, Swor R, Guse C, Brasel K, et al. Does Mechanism of Injury Predict Trauma Center Need? Prehospital Emergency Care. 2011;15(4):518–25.

3. Wigman LD, Van Lieshout EMM, De Ronde G, Patka P, Schipper IB. Trauma-related dispatch criteria for Helicopter Emergency Medical Services in Europe. Injury 2011;42(5):525–33.

## A34 Lessons to be learned from prehospital airway intervention documentation? Are airway intervention documentation templates as successful in-hospital as prehospitally?

### S. Abiks^1^, L. Cutler^2^, K. Monaghan^2^, A. Al-Rais^1^, C. Hymers^2^, R. Bloomer^1^, Y. Kapadia^2^

#### ^1^Department of Anaesthesia, King’s College Hospital - Denmark Hill, London, UK; ^2^Emergency Department, King’s College Hospital - Denmark Hill, London, UK

##### **Correspondence:** S. Abiks (susanabiks@yahoo.co.uk) – Department of Anaesthesia, King’s College Hospital - Denmark Hill, London, UK

Background:

Poor documentation impacts on patient safety, audit, governance and medico-legal process. Prehospital services significantly improved airway intervention documentation by introducing a template (1) and improved data interpretability/comparison by using an Utstein-style template (2). Poor documentation of tracheal intubation (TI) in our emergency department (ED) was an incidental finding in another audit and instigated introduction of a documentation template. Has this intervention been as successful in-hospital?

Method:

Retrospective review of ED documentation of TI following template introduction from August 2014-October 2014.

Results:

83 TI cases were identified, 57.8 % (48/83) had documentation, of which 66.7 % (32/48) utilised the template.

Template use was associated with statistically significant improvement in documentation of: weight (p < 0.0001); assistant name (p = 0.0017); assistant speciality (p < 0.0001); team brief (p < 0.0001), C-spine immobilisation (p < 0.0001); preoxygenation (p < 0.0001); cricoid pressure (p = 0.0002); ventilatory assistance (p = 0.0005); laryngoscope blade (p < 0.0001); maintenance drugs (p = 0.0006).

Discussion:

Our documentation is still disappointingly poor especially regarding patient safety issues such as complications. Template introduction was associated with significant improvement, but arguably was not as successful as prehospitally (1). Contributing factors may include diluted ownership and a mixture of paper and electronic notes.

Un-interpretable comparison to previous audit data is an example of how non-uniformity in data collection hinders analysis. Although no specific College guidelines for TI documentation exist the example of the Utstein-style template used in AIRPORT (2) reveals a consensus that improved data uniformity can be achieved. Perhaps it’s time such a consensus is reached for in-hospital TI documentation?

References

1. Bloomer R, Burns BJ, Ware S. Improving documentation in prehospital rapid sequence intubation: investigating the use of a dedicated airway registry form. Emerg Med J. 2013, *30*(4);324-326

2. Sunde GA, Heltne J-K, Lockey D, et al. Airway management by physician-staffed Helicopter Emergency Medical Services – a prospective, multicentre, observational study of 2,327 patients. Scand J Trauma Resusc Emerg Med, 2015;23:57

## A35 Novel biomarkers in *pre*hospital management of *t*raumatic *b*rain *i*njury (the PreTBI study protocol)

### Sophie-Charlott Seidenfaden^1^, Ingunn S. Riddervold^1^_,_ Hans Kirkegaard^2^, Niels Juul^3^_,_ Morten T. Bøtker^1^

#### ^1^Department of Research and Development, Prehospital Emergency Medical Services, Central Denmark Region, 8200 Aarhus N, Denmark; ^2^Research Center for Emergency Medicine, Aarhus University Hospital, 8000 Aarhus C, Denmark; ^3^Department of Neuro Anesthesiology, Aarhus University Hospital, 8000 Aarhus C, Denmark

##### **Correspondence:** Sophie-Charlott Seidenfaden (soseid@rm.dk) – Department of Research and Development, Prehospital Emergency Medical Services, Central Denmark Region, 8200 Aarhus N, Denmark

Background: Traumatic brain injury (TBI) is the leading cause of death and disability among young adults worldwide. A blow to the head can result in anything from a superficial laceration to severe brain injury. By introducing prehospital biomarker measurements, optimal utilization of known biomarkers and identification of novel biomarkers may aid in 3 important clinical issues in TBI patients: 1) Quick rule-out of intracranial damage in patients who do not benefit from hospital services 2) Fast recognition of cerebral damage, optimal initial treatment and rapid triage of high-risk patients directly to specialized hospital departments and 3) Early knowledge of expected outcome in order to modulate the level of intervention.

Hypotheses:

1. Prehospital rule-out of intracranial lesion in mild TBI patients by use of S100B is feasible and safe
2. Prehospital biomarker measurement can help the identification of TBI patients in antithrombotic therapy in need of urgent neurosurgical intervention
3. Prehospital values of a panel of biomarkers add to prognostication in severely injured TBI patients

Methods: A blood sample will be drawn in the ambulance from all patients suffering TBI. Three prospective, observational studies evaluating the potential of prehospital biomarker measurements are planned,

Primary outcome measures:

1. Sensitivity and specificity of S100B in relation to intracranial lesion
2. Sensitivity and specificity of novel biomakers in relation to need of urgent neurosurgical intervention
3. Prognostic value of a panel of biomarkers

Discussion: If biomarkers can safely and feasibly rule-out intracranial lesions in mild TBI and identify high-risk patients among those in antithrombotic therapy, there is a potential in developing a point-of-care analysis for prehospital use, to reduce hospital admissions and CT examinations. If prehospital biomarker values can optimize in-hospital prognostication of severe TBI patients, neurosurgeons and neuro-intensivists can be guided in the clinical decision-making regarding treatment and level of care.

## A36 Hospital outcomes of traumatic railway incidents: a seven-year observational retrospective study of a major trauma centre

### Alice Gao^1^, Zane Perkins^2^, Gareth Grier^2^, Alex Tzannes^2^

#### ^1^Barts and the London SMD, London, UK; ^2^Institute of Pre-Hospital Care, London’s Air Ambulance, London, UK

##### **Correspondence:** Alice Gao (a.gao@smd11.qmul.ac.uk) – Barts and the London SMD, London, UK

Background

Transport is essential to cities and with many methods available it is unfortunately also a common source of injuries. Railway incidents are particularly devastating, however few studies have described this mechanism of injury.

Objectives: To study the epidemiology, injury pattern and hospital outcomes of patients hit by trains.

Method

A retrospective hand searched review of a physician-led prehospital care trauma database was conducted to identify all patients who were hit by trains in a 7-year period from January 2005 to December 2011. A retrospective review of a major trauma centre database was then conducted to follow up patients who survived to hospital.

Results

302 patients who were hit by trains were identified, 155 (51 %) of whom died at scene in the prehospital setting. Of the 147 that survived to hospital, 86 (59 %) patients went to the major trauma centre in this study. Of this group of patients, the male to female ratio was 3.1:1 and the mean age was 41 years old (range: 17 – 79). The median Injury Severity Score (ISS) was 22 (interquartile range: 9 – 34). The median length of hospital stay was 14 days (interquartile range: 1 – 48.5). 69 (81 %) patients survived to hospital discharge and 15 (17 %) died in hospital. Data in terms of outcomes was missing for 2 (2 %) patients.

Conclusion

This study shows that patients hit by trains that survive to hospital are still generally severely injured with multiple injuries. However 81 % of this group of patients survived to hospital discharge, which shows that despite a devastating mechanism of injury, the survivability once patients arrive at a major trauma centre can be high.

The main limitations of this study are information bias due to the retrospective nature of the study, and missing data in terms of outcomes of patients that went to other hospitals.

## A37 Does taking a third crew member affect the on-scene time of HEMS jobs?

### Nathan Hudson-Peacock^1^, Quentin Otto^1^, Laurie Phillipson^2^, Rik Thomas^2^, Ainsley Heyworth^2^

#### ^1^University of Cambridge, School of Clinical Medicine, Cambridge, UK; ^2^Essex + Herts Air Ambulance Trust, Colchester, UK

##### **Correspondence:** Ainsley Heyworth (ainsley.heyworth@ehaat.org) – Essex + Herts Air Ambulance Trust, Colchester, UK

Background

Essex + Herts Air Ambulance Trust (EHAAT) has a clinical crew size of two (doctor and paramedic). If a third crew member is present they provide the role of a Supervisor (training) or Observer (in training). We aimed to see if taking a third crew member impacted on-scene times (OST). Longer OSTs have been shown to negatively affect outcomes [1,2].

Method

The EHAAT database was used to extract OSTs and crew type from 9/12/14– 29/8/15. Jobs that had an OST ≤ 5 mins or ≥ 90 mins were excluded (stand down or external factors). Normality of data sets was confirmed by Shapiro-Wilk analysis. One-way ANOVA assessed any difference between a supervisor, observer or absent third crew member. Additionally data sets for observers and supervisors were combined to create two and three-person crew data set and means compared using a two-tailed student’s t-test.

Results

976 missions were tasked of which 361 were excluded, leaving 615 for analysis. The mean OST (±95 % C.I.) was 37.4 ± 3.4 mins with a supervisor (n = 72), 34.6 ± 1.7 mins with an observer (n = 342), and 35.3 ± 2.2 mins without a third person (n = 201). One-way ANOVA showed that these were not significantly different (p = 0.38). Mean OST (±95 % C.I.) for any three-person crew was 35.1 ± 1.5 mins, and for a two person crew was 35.3 ± 2.2 mins (p = 0.85), showing no significance.

Conclusions

These data show no significant difference in mean OST for HEMS jobs regardless of crew size. This should reassure HEMS organisations that the benefit of the third crew member, whether as a supervisor or observer, does not come at the cost of longer on-scene times.

References

(1) Harmsen AMK, Giannakopoulos GF, Moerbeek PR, Jansma EP, Bonjer HJ, Bloemers FW. The influence of prehospital time on trauma patients outcome: A systematic review. Injury. 2015 Apr;46(4):602–9.

(2) Feero S, Hedges JR, Simmons E, Irwin L. Does out-of-hospital EMS time affect trauma survival? The American Journal of Emergency Medicine. 1995 Mar;13(2):133–5.

## A38 Does pre-hospital rapid sequence induction affect on-scene time of HEMS jobs?

### Quentin Otto^1^, Nathan Hudson-Peacock^1^, Laurie Phillipson^2^, Ainsley Heyworth^2^, Erica Ley^2^

#### ^1^University of Cambridge, School of Clinical Medicine, Cambridge, UK; ^2^Essex + Herts Air Ambulance Trust, Colchester, UK

##### **Correspondence:** Ainsley Heyworth (ainsley.heyworth@ehaat.org) – Essex + Herts Air Ambulance Trust, Colchester, UK

Background

Essex + Herts Air Ambulance Trust (EHAAT) regularly performs rapid sequence induction of anaesthesia (RSI) in the pre-hospital setting for established indications documented in standard operating procedures [1]. These include, but are not limited to actual or impending airway compromise and optimisation of ventilation. It has been shown that a shorter on-scene time (OST) improves patient outcomes [2,3]. This study aimed to identify whether performing pre-hospital RSI affects OST.

Method

An 8-month retrospective database review (HEMSbase, by Medic One Systems) of attended cases was undertaken. Comparison between patients receiving and not receiving RSI were evaluated against OST. Exclusion criteria constituted cases with multiple patients and where the HEMS crew were stood down en-route or at scene. Cases that had an OST ≤ 5 mins or ≥ 90 mins were excluded as it was decided that extremes of OST were most likely due to factors independent of the RSI. Microsoft Excel was used to analyse the data. Normality of data sets was confirmed using the Shapiro-Wilk test. Mean OSTs for jobs with and without RSI were compared using a two-tailed student’s t-test.

Results

632 single-patient cases with recorded OSTs were identified. 23 jobs were excluded due to OST <5mins or >90mins, leaving 609 jobs for statistical analysis. The mean OST (±95 % C.I.) was 30.9 ± 1.4 mins in jobs where RSI was not performed (n = 423) and 42.5 ± 2.0 mins where RSI was performed (n = 186). Student’s t-test confirmed statistically significant difference between groups (p < 0.001).

Conclusions

In the EHAAT system, cases requiring RSI result in a longer OST. It is likely that reasons for this are multifactorial. Further work with these data is required to identify factors that might improve on-scene time.

References

(1) Cowan GM, Burton F, Newton A. Prehospital anaesthesia: a survey of current practice in the UK. Emerg Med J. 2012 Feb 1;29(2):136–40.

(2) Harmsen AMK, Giannakopoulos GF, Moerbeek PR, Jansma EP, Bonjer HJ, Bloemers FW. The influence of prehospital time on trauma patients outcome: A systematic review. Injury. 2015 Apr;46(4):602–9.

(3) Feero S, Hedges JR, Simmons E, Irwin L. Does out-of-hospital EMS time affect trauma survival? The American Journal of Emergency Medicine. 1995 Mar;13(2):133–5.

## A39 Code red: shock index as a prehospital indicator of massive haemorrhage

### Daniel Banner^1^, Ainsley Heyworth^2^, Erica Ley^2^

#### ^1^University of East Anglia, Norwich, UK; ^2^Essex + Herts Air Ambulance Trust, Colchester, UK

##### **Correspondence:** Ainsley Heyworth (ainsley.heyworth@ehaat.org) – Essex + Herts Air Ambulance Trust, Colchester, UK

Background

In patients with suspected massive haemorrhage a 'code red' pre-alert can be given to the receiving hospital by the Essex and Herts Air Ambulance team in order to receive blood on arrival at hospital. Blood loss is notoriously difficult to estimate. No physiological markers have been verified as indicators at present but Shock Index (SI) has been suggested as a potential indicator of shock. We aimed to establish how well the SI correlates with the HEMS team's decision that a patient requires a ‘code red’ pre-alert and if there is any scope for SI to be used to identify ‘code red’ patients.

Methods

A single centre retrospective cohort study was carried out using data from EHAAT’s database of calls attended over an 8-month period for all trauma patients transported to a Major Trauma Centre (MTC). Patients identified as ‘code red’ were allocated to the case group and all others were placed in the control group. Patients were excluded if they had an intracranial injury or if data was missing, preventing the SI being calculated. Descriptive statistics were calculated for both groups and Mann-Whitney U calculations were used to compare the SI between the two groups.

Results

Descriptive statistics for both groups are described. Mann Whitney U calculations showed that there was a statistically significant difference in SI between the two groups (p = 0.0002). U value was 711.5 and Z value was -3.7.

Conclusion

The results show that in EHAAT’s small population there is a significant difference in SI between major trauma patients and code red patients, with SI being higher in code red patients. Hospital follow up data is required to show a definitive link between SI and massive haemorrhage.

## A40 Air ambulance tasking: how accurate are our current methods?

### Madeleine Benson^1^, Nathan Hudson-Peacock^2^, Tony Stone^3^, Erica Ley^3^, Louise Rosson^3^, Ainsley Heyworth^3^

#### ^1^University of Leicester Medical School, Leicester, LE1 9HN, UK; ^2^University of Cambridge Medical School, Cambridge, CB2 0SP, UK; ^3^Essex and Hertfordshire Air Ambulance, Essex and Hertfordshire Air Ambulance Trust, Essex, UK 2138

##### **Correspondence:** Madeleine Benson (madi.benson@hotmail.co.uk) – University of Leicester Medical School, Leicester, LE1 9HN, UK

**Background**

Correct identification of critically ill and injured patients during the first emergency call is vital to the tasking of air ambulances. Essex and Herts Air Ambulance Trust (EHAAT) is dispatched by flight paramedics based on mechanism of injury (MOI), clinical interrogation (INT) or land ambulance crew request (REQ). This study aimed to evaluate how effective current dispatch methods are, based on accurate identification of appropriate patients and time to dispatch.

**Methods**

A retrospective review of 8 months of dispatch data (December 2014 – August 2015) was undertaken. Appropriate dispatch was defined as the requirement for EHAAT to escort the patient to hospital or to provide advanced medical intervention on-scene. Inaccurate dispatch was when EHAAT was either stood down or left the patient in the care of land ambulance crew.

Dispatch times were compared using one-way analysis of variance (ANOVA). Appropriateness of tasking was compared using the χ^2^ test and statistical significance was adjusted to account for multiple testing.

**Results**

910 EHAAT dispatches were analysed: MOI 43.0 % (n = 391), INT 25.8 % (n = 235) and REQ 31.2 % (n = 284). Appropriate dispatch rates were: MOI 50.9 % (199/391), INT 70.6 % (166/235) and REQ 86.6 % (246/284). INT and REQ were both significantly more accurate than MOI (p < 0.017). Additionally, REQ was significantly more accurate than INT (p < 0.017).

Dispatch methods applied prior to land ambulance crew assessment (MOI + INT) accurately identified 59.7 % of patients, with an overtriage rate of 41.7 % and an undertriage rate of 40.3 %. Mean time to dispatch (in minutes) varied significantly (p < 0.01) between methods MOI 6.1, INT 13.3 and REQ 35.9.

**Conclusion**

Clinical interrogation and crew request are both significantly more accurate than MOI at identifying appropriate patients for air ambulance tasking, although time to dispatch increases correspondingly. Over- and under-triage remains an issue, particularly with MOI and INT.

## A41 Modern trauma burden in a district general hospital

### Beth A Lineham^1^, Matthew J Lee^2^, Martin Gough^3^

#### ^1^Trauma and Orthopaedics, Bradford Royal Infirmary, Bradford, West Yorkshire, UK; ^2^General Surgery, Northern General Hospital, Sheffield, South Yorkshire, UK; ^3^General Surgery, Scunthorpe General Infirmary, Scunthorpe, North Lincolnshire, UK

##### **Correspondence:** Beth A Lineham (bethbrown@doctors.org.uk) – Trauma and Orthopaedics, Bradford Royal Infirmary, Bradford, West Yorkshire, UK

**Background**

Trauma care has been centralised with the establishment of major trauma networks, leading to concentration of resources with established pathways. Major trauma centres (MTC) should be involved in the care of most seriously injured patients, with district general hospitals (DGH) acting as a trauma unit for the less seriously injured, or providing primary stabilising interventions. We set out to assess trauma load in our unit.

**Method**

Data collected by the audit department on trauma presentation to A&E was used to identify the trauma burden. A&E department software was then used to identify cases where trauma-team activation had occurred over a 6-month period. Assessment of whether the patient should have bypassed the DGH was based on triage data. The amount of trauma calls within the hospital was collected separately from the operations centre to ensure the data on trauma presentation was full.

**Results**

Based on the A&E data, in the study period there were 64 patients with major trauma, of whom 5 patients (7 %) had a trauma call within the 6-month period. 7 patients (10 %) met the criteria for bypass to a MTC. 4 patients (6 %) were transferred to a MTC.

There were 33 trauma calls in the study period. Only four (6 %) of these patients were included in the A&E trauma report data. This suggests that current mechanisms for recording trauma workload are insufficient.

**Discussion**

Our DGH sees a low volume of trauma, but 10 % of this should be triaged direct to the MTC. We suspect the current system does not record trauma activations accurately. To completely assess our trauma workload we plan to undertake a full prospective audit of patients attending A&E. Discussion of findings with paramedic colleagues should be undertaken to identify pre-hospital triage issues.

## A42 Establishing a legal service for major trauma patients in two UK major trauma centres

### William H Seligman^1^, Hannah E Thould^1^, Andrew Dinsmore^2^, Charlotte Tan^2^, Julian Thompson^1^, C Andy Eynon^3^, David J Lockey^1^

#### ^1^North Bristol NHS Trust, Bristol, UK; ^2^Stewarts Law LLP, London, UK; ^3^University Hospital Southampton NHS Foundation Trust, Southampton, SO16 6YD, UK

##### **Correspondence:** William H Seligman (seligmanw@gmail.com) – North Bristol NHS Trust, Bristol, UK

**Background**

Major trauma causes unanticipated critical illness and patients have often made few arrangements for what are sudden and, in many cases, life-changing circumstances. This can lead to significant financial, housing, insurance, legal and employment issues for patients and their families.

A national law firm, Stewarts Law LLP, worked with the major trauma services to develop a free and comprehensive legal service^1^ for major trauma patients and their families at two Major Trauma Centres (MTCs).

**Methods**

In 2013, a legal service was established for major trauma patients at North Bristol and University Hospital Southampton NHS Trusts. Referrals are usually made by trauma nurse practitioners and it operates within a strict ethical framework. [1] A retrospective analysis of the activity of this legal service between September 2013 and October 2015 was undertaken.

**Results**

129 major trauma patients and their families (and 10.8 % of 854 major trauma ICU admissions) were seen by the legal teams at the two MTCs. 841 hours of free legal advice were provided on non-compensation issues, including welfare, grants and benefits (88 % of referrals), insurance (72 %) and financial issues (53 %). In 70 % of cases, potential compensation claims were also identified. Consultations are usually conducted in hospital and initial feedback from participants has been positive.

**Discussion**

This initiative demonstrates a need for early legal advice for major trauma patients and their families to address a range of non-compensation issues. Early identification and management of these issues can help to alleviate the anxiety experienced by patients and their families and may perhaps assist with recovery and rehabilitation. The utilisation of this service suggests that it should be considered at other MTCs.

**Reference**

1. Eynon, C. Andy, Andrew Dinsmore, and Stuart Dench. "Lawyers in intensive care: introducing an acute legal service for patients with critical illness." *Journal of the Intensive Care Society* 11.2 (2010): 109-111.

## A43 Prehospital assessment and care of patients – a study of the use of guidelines when assessing head trauma

### Rebecka M Rubenson Wahlin^1^, Veronica Lindström^1,2^, Sari Ponzer^1^, Veronica Vicente^1,2^

#### ^1^Department of Clinical Science and Education, Södersjukhuset, Karolinska Institutet, Stockholm, Sweden; ^2^Academic EMS, Stockholm, Sweden

##### **Correspondence:** Rebecka M Rubenson Wahlin (rebecka.rubenson.wallin@ki.se) – Department of Clinical Science and Education, Södersjukhuset, Karolinska Institutet, Stockholm, Sweden

**Background:** Best evidence guidelines for prehospital management of traumatic brain injuries (TBI) have been developed and established in several countries. These guidelines are intended to standardize assessment and treatment of patients with head trauma and by doing so to improve outcomes for TBI patients.

The aim of this study was to study to what extend Prehospital Emergency Care Nurses (PECN) followed the guidelines and also documented their assessment and management of patients with head trauma.

**Method:** A retrospective observational study was conducted by evaluating 2789 prehospital medical records on adult patients in Stockholm, Sweden during year 2012.

**Results:** The results showed that nearly 80 percent of the patients received at least one intervention by the PECNs and that males were given higher transport priority but also that females received significantly more analgesics. The assessment documented by the PECNs was not optimal concerning documentation of the Glasgow Coma Scale but the documented assessment of circulation and especially respiratory rate was high when compared to previous studies.

**Conclusion:** The findings of this study should be seen as a step forward in developing the prehospital care delivered by the PECNs in the ambulance services.

**References**

1. Bellander BM, Sollid S, Kock-Jensen C, Juul N, Eskesen V, Sundstrom T, Wester K, Romner B: Prehospital management of patients with severe head injuries. Scandinavian guidelines according to Brain Trauma Foundation (In Swedish). *Lakartidningen* 2008, 105(24-25):1834-1838.

2. Watts DD, Hanfling D, Waller MA, Gilmore C, Fakhry SM, Trask AL: An evaluation of the use of guidelines in prehospital management of brain injury. *Prehospital emergency care : official journal of the National Association of EMS Physicians and the National Association of State EMS Directors* 2004, 8(3):254-261.

3. Laudermilch DJ, Schiff MA, Nathens AB, Rosengart MR: Lack of emergency medical services documentation is associated with poor patient outcomes: a validation of audit filters for prehospital trauma care. *Journal of the American College of Surgeons* 2010, 210(2):220-227.

## A44 An audit of pre-hospital blood pressure management resulting from head injury

### Pamela Eligio^1^, Anthony Hudson^2^

#### St George's University of London, London, UK

##### **Correspondence:** Pamela Eligio (m1201555@sgul.ac.uk) – Emergency Department, St George’s Hospital, London, London, UK

**Background**

Where rapid sequence intubation (RSI) is indicated, pre-hospital management of isolated head injury relies on control of hypotension. Kent, Surrey and Sussex air ambulance (KSS) standard operating procedures (SOPs) require systolic blood pressure (SBP) ≥ 100 mmHg, and the Association of Anaesthetists of Great Britain and Ireland and National Institute for Health and Care Excellence stipulate national guidelines of mean arterial pressure (MAP) > 80 mmHg [1,2]. This audit aims to: (1) assess KSS adherence to SOPs and (2) compare these results against national guidelines.

**Method**

Analysis of KSS data for adults ≥ 18 years who required RSI due to isolated head injury, with transfer to hospital by helicopter.

**Results**

44 patients were identified with a total of 1,070 instances of recorded SBP. Of these, only 12 patients had SBP readings for every instance of recorded observations. KSS SOPs show 41 % of patients had at least one episode of hypotension during transfer (6 % of total instances) compared to 59 % for national guidelines (14 % of total instances).

**Discussion**

KSS failed to adhere to SOPs for 41 % of patients and to national guidelines for 59 %. Increased instances of hypotensive episodes suggest that MAP is a better measure in real terms. The availability of data reflects the challenge of pre-hospital care and may be improved using upgraded technology or invasive methods such as arterial lines.

Recommendations: (1) KSS SOPs to include MAP and (2) review SBP measurement to improve the efficacy of patient management in the pre-hospital care setting.

**References**

[1] National Institute for Health and Care Excellence. Head injury. https://www.nice.org.uk/guidance/cg176/evidence/full-guideline-191719837. Accessed 10 Jul 2015.

[2] The Association of Anaesthetists of Great Britain and Ireland. Recommendations for the safe transfer of patients with brain injury. https://www.aagbi.org/sites/default/files/braininjury.pdf. Accessed 12 Jan 2015.

## A45 The surgical contribution of surface shading volumetric rendering techniques in rib fracture management

### Robert Young, Dimitri Amiras, Ian Sinha

#### Major Trauma Unit, St Mary’s Hospital, Imperial College London, London, UK

##### **Correspondence:** Robert Young (robertanthonyyoung@gmail.com) – Major Trauma Unit, St Mary’s Hospital, Imperial College London, London, UK

Background

Blunt thoracic trauma a significant cause of injury in the civilian setting, and surgical rib fracture fixation is becoming an increasingly common form of management. In select patient groups with flail chest pathology, surgical fixation has shown to reduce ventilation requirements, length of ITU stay and associated complications; proving cost effective despite the initial additional costs of surgery.

Method

The best outcomes have been shown in patients who receive early surgical intervention, however, the conventional investigations just as plain chest X-ray and standard CT have low sensitivity for flail pathology identification. Newer techniques of surface shading volumetric rendering CT not only increase diagnostic sensitivity for rib fractures and flail segments, but additionally provide the operating surgeon with vital information on the surrounding anatomy. This aids the choice of incision site and surgical approach in an area less familiar to many surgeons, due to a long tradition of conservative management in blunt thoracic trauma and flail chest.

Results

Here we present the images derived though surface shading volumetric rendering techniques in a patient who suffered blunt thoracic trauma and subsequently underwent successful surgical rib fixation.

Discussion

These illustrate the superior anatomical detail achieved over standard CT axial images, valuable in process of both diagnosis and interventional surgical management.

